# Pelvis perturbations in various directions while standing in staggered stance elicit concurrent responses in both the sagittal and frontal plane

**DOI:** 10.1371/journal.pone.0272245

**Published:** 2023-04-12

**Authors:** Michelle van Mierlo, Jean A. Ormiston, Mark Vlutters, Edwin H. F. van Asseldonk, Herman van der Kooij

**Affiliations:** 1 Department of Biomechanical Engineering, University of Twente, Enschede, The Netherlands; 2 Department of Research, Sint Maartenskliniek, Nijmegen, The Netherlands; 3 Department of Biomechanical Engineering, Delft University of Technology, Delft, The Netherlands; University of Innsbruck, AUSTRIA

## Abstract

Increasing knowledge on human balance recovery strategies is important for the development of balance assistance strategies using assistive devices like a powered lower-limb exoskeleton. One of the postures which is relevant for this scenario, but underexposed in research, is staggered stance, a posture with one foot in front. We therefore aimed to gain a better understanding of balance recovery in staggered stance. We studied balance responses at joint- and muscle levels to pelvis perturbations in various directions while standing in this posture. Ten healthy individuals participated in this study. We used one actuator beside and one behind the participant to apply 150 ms perturbations in mediolateral (ML), anteroposterior (AP) and diagonal directions, with a magnitude of 3, 6, 9 and 12% of the participant’s body weight (BW). Meanwhile, motion capture, ground reaction forces and moments, and electromyography of the muscles around the ankles and hips were recorded. The perturbations caused movements of the centre of mass (CoM) and centre of pressure (CoP) in the direction of the perturbation. These were often accompanied by motions in a direction different from the perturbation direction. After perturbations perpendicular to the line between both feet, large and significant AP deviations were present of the CoM (-0.27 till 0.40 cm/%BW, p < 0.029) and CoP (-0.99 till 0.80 cm/%BW, p < 0.001). Also, stronger responses on joint and muscle level were present after these perturbations, compared to AP and diagonal perturbations collinear with the line between both feet. The hip, knee and ankle joints contributed differently to the balance responses after the different perturbation directions. To conclude, standing in a staggered stance posture makes individuals more vulnerable to perturbations perpendicular to the line between both feet, requiring larger responses on joint level as well as contributions in the sagittal plane.

## Introduction

Research into human biomechanics, and in particular human balance recovery, is vital to the development of technologies supporting rehabilitation of individuals with movement disorders. A specific example of such a supporting technology is a powered lower-limb exoskeleton, enabling the possibility to stand and walk for spinal cord injury patients. In order to assist balance in this situation, extensive knowledge is required on human balance recovery strategies. One of the postures that is underexposed in research regarding human balance is staggered stance, a posture with one foot in front. Maybe unconsciously, but it is also a posture that healthy individuals adapt in various daily-life situations. For example, to counteract disturbances during travelling in a train or during sports. The knowledge on how to maintain balance in staggered stance is important for walking with a lower-limb exoskeleton. Currently, most exoskeletons have a very slow walking speed (about 0.26 ms^-1^ [1–4]), which often results in a non-dynamical gait pattern with prolonged double support stance phases. In the case a following step is not being triggered by the user or exoskeleton this can result in a staggered stance posture. Increasing understanding of human balance control strategies while standing in staggered stance can help in the development of balance assistance in these kind of scenarios.

Staggered stance is a hybrid of tandem and parallel stance. In parallel stance, where the feet are next to each other, unperturbed sagittal plane balance is dominated by modulations of the ankle moments. For the frontal plane hip ab- and adduction moments realize the major contribution to balance. Contrarily, during quiet tandem stance, in which one foot is in front of the other, the hips account for the dominant balance response in the sagittal plane through flexion and extension [5]. Mediolateral (ML) stability during tandem stance is predominantly governed by ankle in- and eversion with a smaller contribution from the hip ab- and adduction [6]. While standing in this posture, naturally most weight is put on the trailing leg, resulting in a stronger muscle contribution from this leg compared to the leading leg [7].

Standing in a staggered stance posture combines several of the balance responses and sensitivities of the tandem and parallel stances [5, 6, 8]. It also allows for more possibilities to recover balance in both the sagittal and frontal plane, compared to the tandem or parallel stance, which often has stronger mechanical constraints in either of the planes [5]. A study on postural sway during unperturbed staggered stance showed the dependency between balance mechanisms performed in the frontal and sagittal plane. For balance in the sagittal plane this meant that the ankle joint even had to cancel out a counteracting balance mechanism performed by the hip joint [6]. This is unlike the frontal plane, were the hip and ankle joint reinforce each other to maintain balance [6, 8, 9]. Overall, for a staggered stance posture with most of the weight on the trailing leg, individuals tend to use balance strategies similar to those used in parallel stance [5], while CoP variability increases with respect to parallel stance [6, 8].

The ability to maintain balance is largely dependent on the size and orientation of the base of support (BoS) [6, 10]. A larger BoS allows for a larger displacement of the centre of pressure (CoP). This is one of the mechanisms that has shown to be effective in controlling the centre of mass (CoM), for example during double support [11, 12]. Another strategy will be used when the BoS is small, being a counter-rotation mechanism induced by upper body movement to change the orientation of the ground reaction force (GRF) [11, 13]. Coordinated hip, knee and ankle joint moments can redirect the GRF and modulate the CoP position in order to control the linear and angular momentum of the whole body [14]. The staggered stance posture, with a large anteroposterior (AP) BoS, will allow for an effective use of the ankle strategy in the sagittal plane and for a large AP weight shift. On the other hand, maintaining balance in the frontal plane might be more challenging in this posture, since there is limited space for CoP modulation and weight shift in ML direction.

Extensive research has been done to analyze human balance recovery in parallel or tandem stance after balance perturbations such as: multi-directional surface translations [13, 15–17], external forces applied to the pelvis [9, 18–22], visual perturbations [8] and self induced perturbations [23]. These studies give insights into the use of the hip, knee and ankle joints in order to maintain balance. The studies by Henry et al. [15] and Matjacic et al. [18] showed that, while standing in a parallel stance, diagonal perturbations provoke the largest joint and muscle responses out of all applied perturbation directions. This large response is caused by a combination of the responses to an AP and ML perturbation [15, 18]. Various studies also showed that responses in both the frontal- and sagittal plane can be observed after perturbations in only a single plane [8, 15, 18, 23]. For example tibialis anterior and rectus femoris activity after ML perturbations, adductor longus activity after anterior perturbations [15], or an increased CoP variability in a plane perpendicular to the plane of the perturbation [8]. Lee et al. [23] showed a dependency of the postural adjustments on the stance posture as well. They showed that individuals had larger ML CoP displacement while standing in staggered stance, compared to parallel stance following self-induced backward perturbations [23]. These studies have shown a coupling in perturbations and responses between the frontal- and sagittal plane. However, it is not clear yet how balance recovery strategies in both planes are used after perturbations from different directions while standing in staggered stance.

In this study we aim to establish how hip, knee and ankle joints responses contribute to and co-operate in balance recovery after pelvis perturbations in various directions while standing in a staggered stance. Since the size and orientation of the BoS largely affect the risk of losing balance, the staggered stance posture influences the sensitivity to certain perturbation directions. Individuals will be more vulnerable to perturbations in the direction where the BoS is the smallest, which are in the directions perpendicular to the line between both feet. It is hypothesized that motions in the sagittal plane will play an important role after all perturbation directions. Because of the staggered stance posture there will be a coupling between the motions in both the sagittal and frontal plane, allowing for balance recovery contributions from the sagittal to the frontal plane and vice versa. Since the BoS in the sagittal plane is the largest, the joints can induce larger modulations in the CoP and horizontal GRF, facilitating an efficient recovery of the CoM.

## Materials and methods

### Participants

This research was approved by the EWI/ET Ethics committee of the University of Twente under reference number RP 2019–88. All participants gave written informed consent in accordance with the Declaration of Helsinki before participating in the study. Ten participants, five female and five male, with no known history of neurological, muscular or orthopedic problems participated in this study. The participants had an average (±SD) age of 23.6 ± 2.9 years, height of 1.76 ± 0.05 m, leg length of 0.91 ± 0.06 m measured from the ground to the trochanter major and weight of 69.8 ± 7.8 kg.

### Setup

**Fig 1:**
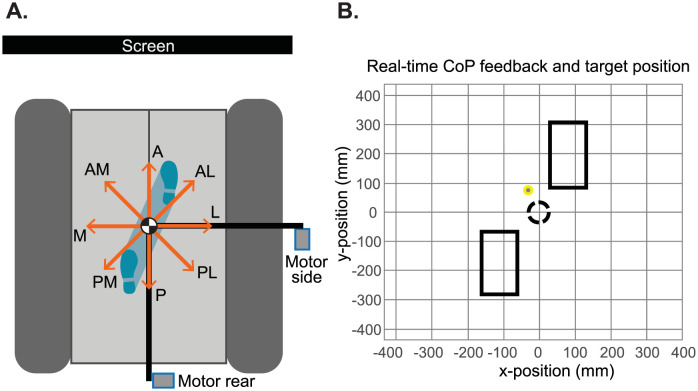
Experimental setup. A) Schematic top-down view of the setup, with one motor (side) placed to the right of the participant, and the other motor (rear) placed posterior of the participant. The orange arrows indicate the 8 perturbation directions. A = anterior, P = posterior, M = medial, L = lateral, AM = anterior-medial, AL = anterior-lateral, PM = posterior-medial, PL = posterior-lateral. A screen is placed directly in front of the participant displaying the position of their feet and CoP as well as the desired CoP position in real-time. B) Feedback presented on the screen. Top-down view of the participant’s feet depicted with the rectangles, based on the position data of the 1^st^ and 5^th^ metatarsi together with the calcanei. The yellow dot presents the participant’s CoP. The black dashed circle is the desired CoP position.

The experiments were carried out on a split-belt treadmill (custom YMill, Motek medical, Culemborg, The Netherlands), with the belts standing still. Two force plates were present beneath the belts for the measurement of GRFs and moments. Two motors (SMH60, MOOG, Nieuw-Vennep, The Netherlands) were placed on the rear and side of the treadmill, see the top-down view in Fig 1a. The participants wore a modified universal hip abduction brace (Distrac Wellcare, Hoegaarden, Belgium; weight 1 kg) which was attached to the motors via horizontal carbon rods and a lever arm of 0.3 m. Load cells (Model LR350 FUTEK, Los Angeles, CA, USA) where positioned in the horizontal rods, to measure and control the applied forces. The motors were controlled via a main computer (Linux, Ubuntu 16.04 LTS) and seven secundary devices: four Beckhoff modules (three analog input and one analog output, Beckhoff Automation GmbH, Germany), Haptic control unit (Moog PC CB79047–401_HCU, Nieuw-Vennep, The Netherlands) and two motor drives (Moog MSD 3200 Servo Drive, Nieuw-Vennep, The Netherlands). An admittance controller was used to minimize the interaction forces during standing and to track the desired forces at the moment a perturbation was given. A detailed description of this controller can be found in [24]. A screen was positioned in front of the treadmill, which was used to give the participant feedback on the position of their CoM and feet as well as the desired CoM position (see section ‘Experimental protocol—Control position centre of mass’ for more details).

### Data collection

Kinematic marker data was acquired using an 8-camera infrared motion capture system (Oqus 600+, Qualysis, Götenborg, Sweden). The data was recorded at 128 Hz with the Qualysis Track Manager software (QTM, Qualysis, Götenborg, Sweden). In total six marker clusters were used on: the right and left shank and thigh, the sternum and on the front of the pelvis brace. Twenty-three individual markers were placed on bony landmarks using double-sided tape: on the 7^th^ cervical vertebra and the right and left calcaneus, 1^st^ and 5^th^ metatarsal heads, toes, medial and lateral malleoli, medial and lateral epicondyles of the femur, anterior and posterior superior iliac spine and acromia. The analog data measured by the force plates and EMG electrodes (Delsys Bagnoli, Natrick, USA) were recorded via an analog interface (Kistler 5695A DAQ) at 2048 Hz, synchronised with the motion capture data. Twelve wired surface EMG electrodes were placed according to the Seniam guidelines on the following muscles of the right and left leg: gluteus medius (mGMe), gluteus maximus (mGMa), adductor magnus (mAM), soleus (mSOL), tibialis anterior (mTA) and peroneus longus (mPL) [25]. The interaction forces between the participant and the motors were recorded at 1000 Hz, via the computer controlling the motors. This computer was also sending a synchronisation signal, which was recorded via the analog interface, to synchronise the kinematic data with the forces.

### Experimental protocol

#### Participant preparation

The reflective markers and marker clusters were attached to the participant, along with the wired EMG electrodes. Before starting the experiment the maximum voluntary contraction (MVC) was recorded, by performing a muscle-specific exercise for each individual muscle. The participant wore a safety harness (Honor, FBH-10) to prevent injury in case of a fall.

#### Staggered stance posture

During the experiment, participants stood still on the treadmill in a staggered stance, with the right foot in the leading position. The locations where the participant had to place their feet were marked on the treadmill. The step width and length were based on the average step width (0.15 m) and length (0.40 m) during walking at 0.5 ms^-1^ [26]. These measures were scaled with a factor determined by the participant’s leg length (*l*) with respect to the average leg length *l*_*av*_ = 0.91 *m*, based on a comparable population from Wu et al. [26]: *Scale* *factor* = *l*/*l*_*av*_. This walking speed was selected to better relate the results to come to a halt in the double support phase of very slow walking. Participants were asked to stand up straight with the arms crossed over their torso to prevent contributions of arm swing to the balance recovery and to avoid collision between the participant’s arm and the rod on the side. Participants were also requested to stand with their knees slightly bent to prevent locking of the knee joint. They were instructed to refrain from stabilizing themselves by using the bars on the sides of the treadmill, unless this was really necessary and a side step did not suffice.

#### Control position centre of mass

Since the participants were standing still, we used the CoP location as a representation of the particpants’s CoM during the experiment. To control the initial posture of the participant, perturbations were only given when the participant’s CoP was within a certain target position for at least 3 s. In order to assume this position, the participants received feedback of their CoP and feet position as well as the desired CoP position via the screen in front of them. To generate this feedback the force plate and marker data were used in real time via a connection between the QTM SDK and a Python GUI. Fig 1b shows a screenshot of what the participants saw on the screen, representing a top-down view of the positions of the feet and CoP. The desired CoP position approximates the halfway point during the double support phase, when the participants shift their weight from the trailing foot to the leading foot. A margin with a radius of 3 cm was taken around this point and displayed as a circle on the screen.

#### Perturbations

Pelvis perturbations were given in 8 different directions: anterior (A), posterior (P), medial (M), lateral (L) and diagonally anterior-medial (AM), anterior-lateral (AL), posterior-medial (PM) and posterior-lateral (PL), shown in Fig 1a. The perturbations were given at 4 different magnitudes (3%, 6%, 9% and 12% of the participant’s body weight (BW)) and lasted for 150 ms. The perturbations were given when the participant’s CoM was within the target for a random time between 3 and 5 s. Each participant performed 8 trials, containing each unique perturbation (direction and magnitude) once in a randomised order (8 x 4 = 32 perturbations per trial). In total this resulted in 256 perturbations per participant.

### Data processing

#### Pre-processing

Using the QTM software the recorded marker trajectories were labeled and all missing samples were filled with the polynomial gap filling tool. Further processing was done with Matlab (2022a, MathWorks). The marker and force plate data were filtered with a zero phase 4^th^ order 10 Hz low pass Butterworth filter. OpenSim 4.2 was used to scale the generic 23 segment model (gait2392) for each participant [27]. The inverse kinematics, analyze and inverse dynamics tools of OpenSim were used to obtain the joint torques, and positions and velocities of both the CoM of each segment and the total body. GRFs and moments were used to calculate the position of the CoP. The EMG amplifier (Delsys) had a build in filter, filtering the data to a 20–450 Hz bandwidth. The data was detrended by subtracting the mean. This was followed by a zero phase 1^st^ order 48–52 Hz Butterworth bandstop filter, rectification and zero phase 2^nd^ order 10 Hz Butterworth low-pass filter. The EMG data of each muscle was normalized to the maximum value of the corresponding muscle recorded during the MVC.

#### Data selection

As we were interested in assessing balance recovery strategies with the feet in place, all balance responses that involved taking a step were removed from the data (the participants were unaware of this). A step was identified when all of the following events were detected: 1) After a perturbation, for a duration of at least 0.04 s the vertical component of the GRF of one of the belts was lower than the threshold value of 20 N; 2) There was a change of at least 0.05 m of the toe and 5^th^ metatarsus marker of the corresponding foot.

#### Outcome measures

To indicate the balance sensitivity to the different perturbation magnitudes and directions, the number of repetitions that required a step for each perturbation condition (magnitude and direction) were counted and expressed as the percentage of the total number of repetitions of the respective condition. To quantify the rate of the response, the time to the point of return was defined. This was expressed as the time from the instant the perturbation started until the CoM velocity in both the AP and ML directions was directed towards the starting position. A range of the first 1.5 s after the start of a perturbation was selected for the following outcome measures: CoP position, CoM position, EMG activity, and joint moments. To determine the maximum deviation of the CoP and CoM position for both the AP and ML directions, the largest deviation with respect to their starting position were considered within the 1.5 s window. For the EMG activity and joint moment outcome measures the mean value was taken over this range. Averages have been taken over the 8 repetitions of each perturbation condition (direction and magnitude) within each participant, followed by an average across all participants. Baseline measures were taken for the EMG activity and joint moments over 1 s before the start of the perturbation. For the baseline value averages have been taken over all repetitions within each participant, followed by an average across all participants.

### Statistics

The effect of the perturbations on the various outcome measures was assessed with linear mixed models. This analysis was performed in R4.2.0 (R Core Team, 2021, Vienna, Austria). Linear mixed models were fitted based on a maximum-likelihood estimation for the following outcome measures: maximum deviation of AP CoM, ML CoM, AP CoP and ML CoP with respect to the initial position, mean EMG of the mSOL, mTA, mPL, mGMa, mGMe and mAM of the leading (right) and trailing (left) leg, and the mean joint moments of the ankle in-/eversion and plantar-/dorsiflexion, knee flexion/extension, hip flexion/extension and ab-/adduction and lumbar bending and extension over the first 1.5 s after the start of the perturbation. The final model structure was selected based on the AIC, BIC and likelihood-ratio test [28]. The best model fit included the perturbation magnitude and direction as a fixed effect together with their interaction and random effects for the intercept and slope, to take into account the participant effects. The perturbation magnitude was added as a continuous variable and the perturbation direction was added as a categorical variable. The residuals of the fit were checked for normality and heteroscedasticity. The main effects were tested with a significance level of α = 0.05 using the Wald t-test with a Kenward-Roger correction for the degrees of freedom.

## Results

### Effect perturbations

**Fig 2:**
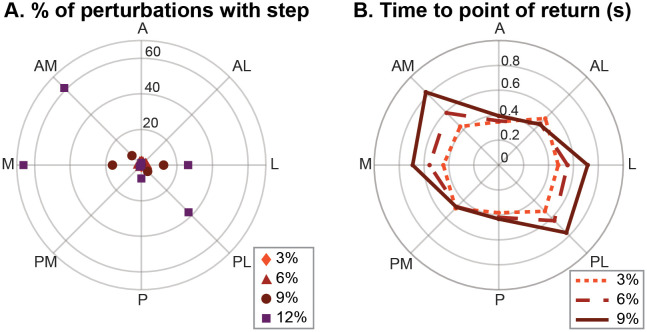
Effect perturbation on stepping and time to point of return. All based on group averages. A) The percentage of the perturbations in each direction and magnitude after which a step was required. B) Time needed to reach the point of return for each perturbation magnitude and direction.

On average across all participants, the staggered stance posture resulted in a weight distribution of 60.3±4.2% on the trailing leg. In the following sections the M, L, AM and PL perturbation directions will be referred to as ‘perturbations approaching perpendicularity to the line between both feet’ and ‘perturbations approaching collinearity with the line between both feet’ will be used for perturbations in the directions A, P, AL and PM, see Fig 1a for the visualisation of these directions. After strong perturbations perpendicular to the line between both feet, participants had to take a step to recover balance more often compared to the perturbations approaching collinearity with the line between both feet. For the 12% magnitude perturbations in the directions M and AM this was in more than 60% of the perturbations, Fig 2a. Since this resulted in too few data samples compared to the other magnitudes, together with remaining repetitions not showing consistent responses because of the extreme perturbation magnitude, this magnitude was removed from the rest of the analysis. In addition, the analysis of the time to the point of return demonstrated that the participants were challenged most by the perturbations perpendicular to the line between both feet. The time to the point of return increased with the perturbation magnitude, up to 0.8 s for the strongest perturbations in these directions, Fig 2b. This effect was less present for the perturbations collinear with the line between both feet, for which the time to the point of return was around 0.4–0.5 s. For all perturbations it holds that after the the end of the perturbation it took some extra time before the CoM started to move in the direction of the starting position.

**Fig 3:**
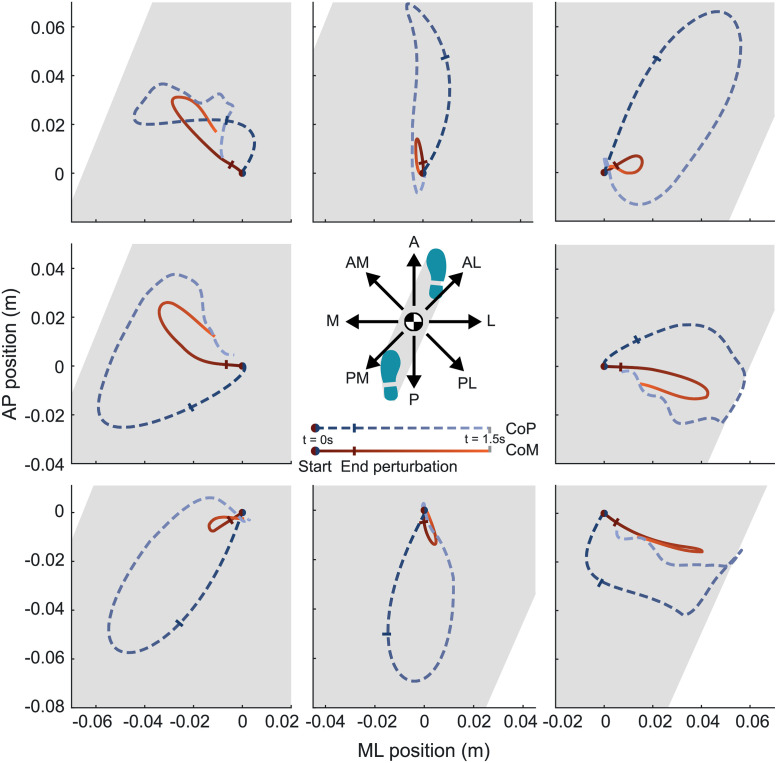
Top-down view CoM and CoP. Top-down view of the centre of mass (CoM in red) and centre of pressure (CoP in blue) trajectories after a 9% magnitude perturbation given in the direction indicated in the middle of the figure. A trajectory of 1.5 s is presented, with the start of the perturbation (t = 0 s) indicated with a dot and the end of the perturbation with a perpendicular line. The shaded area indicates the base of support. The presented results are the averages across all participants.

The perturbations directly induced a motion of the CoM in the direction of the perturbation, Fig 3. If the perturbations were perpendicular to the line between both feet the total CoM deviations were larger compared to those after perturbations collinear with the line between both feet. For almost all perturbation directions, increasing the perturbation magnitude significantly affected the CoM deviation (ranging from -0.41 to 0.52 cm/%BW) in both the ML and AP direction (p always < 0.036), Table 1. This means that the perturbation also affects the plane perpendicular to the perturbation plane. This was the case for all except A perturbations, after which the CoM deviation did not significantly change in ML direction (p = 0.423) and for AL perturbations, after which the CoM deviation did not significantly change in AP direction (p = 0.115), both as result of an increasing perturbation magnitude. Detailed outcomes in the form of time series and complete results of the statistical tests can be found in the S1 File and S2 File. Especially for perturbations in ML direction, a clear response was present in the sagittal plane in terms of CoM and CoP positioning. After M perturbations the CoM was brought more forward (0.30 cm/%BW, p = 0.029), while after L perturbations the CoM was brought backward (-0.20 cm/%BW, p < 0.001) with respect to the unperturbed condition. Generally the CoP followed a trajectory surrounding the CoM, allowing to steer the CoM back to the starting position. After the perturbations collinear with the line between both feet the CoP trajectory made a large deviation, making use of the size of the BoS and enabling the quick return towards the initial condition as shown in Fig 2. For the perturbations perpendicular to the line between both feet the CoP trajectory was limited by the BoS boundaries.

**Table 1:** Parameter estimates.

	Deviation (cm)				Normalized EMG											
	AP		ML		Trailing leg						Leading leg					
	***CoM***	***CoP***	***CoM***	***CoP***	***Sol***	***TA***	***PL***	***GMa***	***AM***	***GMe***	***Sol***	***TA***	***PL***	***GMa***	***AM***	***GMe***
***Intercept***	-0.14	0.31	0.13	0.11	**0.123**	0.015	**0.020**	**0.054**	**0.021**	**0.042**	**0.071**	**0.020**	**0.036**	**0.083**	**0.027**	**0.049**
***Mag***	**0.16**	**0.84**	-0.02	-0.03	0.003	0.001	0.002	0.000	0.000	0.000	0.001	0.002	0.002	0.001	0.000	0.001
Mag*Dir-AL	-0.11	-0.08	**0.20**	**0.68**	-0.003	**0.007**	**0.005**	0.000	0.001	0.000	0.001	**0.004**	0.001	0.001	0.000	**0.002**
Mag*Dir-L	**-0.36**	**-1.45**	**0.54**	**0.89**	-0.003	**0.028**	**0.018**	**0.001**	**0.005**	**0.002**	**0.004**	**0.018**	**0.011**	**0.003**	0.001	**0.006**
Mag*Dir-PL	**-0.43**	**-1.83**	**0.50**	**0.87**	-0.003	**0.029**	**0.019**	**0.001**	**0.005**	**0.002**	**0.004**	**0.018**	**0.010**	**0.004**	**0.002**	**0.006**
Mag*Dir-P	**-0.31**	**-1.63**	**0.07**	0.06	-0.002	0.006	**0.004**	0.000	0.000	0.000	-0.001	0.001	-0.001	0.000	0.000	0.001
Mag*Dir-PM	**-0.24**	**-1.53**	**-0.12**	**-0.59**	0.003	0.001	0.001	0.000	0.000	0.001	-0.001	-0.001	-0.002	0.000	0.001	0.000
Mag*Dir-M	**0.14**	**-0.37**	**-0.39**	**-0.66**	**0.015**	**0.006**	**0.005**	0.000	**0.002**	**0.001**	**0.004**	0.003	**0.005**	-0.001	**0.004**	0.001
Mag*Dir-AM	**0.24**	-0.04	**-0.32**	**-0.54**	**0.014**	0.005	**0.005**	0.000	**0.002**	**0.001**	**0.004**	0.003	**0.005**	0.000	**0.003**	0.001

	Joint moment (Nm)											
	Lumbar		Trailing leg					Leading leg				
	***Bending***	***Flexion***	***Hip ab-/adduction***	***Ankle in-/eversion***	***Hip flex-/extension***	***Knee flex-/extension***	***Ankle plantar-/dorsiflexion***	***Hip ab-/adduction***	***Ankle in-/eversion***	***Hip flex-/extension***	***Knee flex-/extension***	***Ankle plantar-/dorsiflexion***
***Intercept***	**2.782**	**17.815**	**-13.594**	**12.001**	**16.501**	**10.805**	**-31.267**	**-5.138**	-0.041	**-18.998**	0.422	**-8.073**
***Mag***	0.107	-0.145	0.054	0.057	0.021	**-0.255**	**-0.351**	-0.112	-0.003	-0.114	**-0.213**	**-0.351**
Mag*Dir-AL	-0.178	0.101	0.165	**-0.231**	0.069	**0.492**	**0.956**	**-0.218**	0.005	-0.035	**0.684**	**0.675**
Mag*Dir-L	**-0.823**	0.258	**0.293**	**-0.525**	**0.543**	**1.469**	**2.378**	-0.146	-0.057	**-0.541**	**0.909**	**1.434**
Mag*Dir-PL	**-0.889**	0.287	0.009	**-0.520**	**0.585**	**1.566**	**2.379**	-0.061	-0.045	**-0.497**	**0.722**	**1.344**
Mag*Dir-P	**-0.234**	0.364	**-0.266**	**-0.134**	**0.165**	**0.696**	**0.870**	0.138	0.001	-0.028	0.183	**0.570**
Mag*Dir-PM	**-0.250**	0.204	**-0.621**	**0.137**	-0.057	0.098	**-0.299**	**0.267**	0.015	0.218	-0.140	**0.273**
Mag*Dir-M	**0.462**	**0.916**	-0.102	**0.281**	**-0.200**	**-0.368**	**-1.383**	**0.358**	0.063	**-0.391**	**-1.098**	**-0.689**
Mag*Dir-AM	**0.484**	**0.873**	0.148	**0.240**	**-0.237**	**-0.540**	**-1.288**	**0.250**	0.059	**-0.475**	**-0.993**	**-0.771**

The parameter estimates for the intercept, perturbation magnitude (Mag) and interaction between perturbation magnitude and direction (Mag*Dir) for each model. Parameters with a significant contribution (p < 0.05) are shown bold and underlined. The color indicates whether the overall slope (combining the parameter for perturbation magnitude and the interaction) for a certain direction is positive (orange) or negative (blue). The default perturbation direction is A, therefor the interaction is not shown separately. The parameters for the direction are not being presented, since these did not significantly contribute, with only a few exceptions. Which is as expected, since we kept the initial posture similar over the repetitions and directions.

### Muscle level response

**Fig 4:**
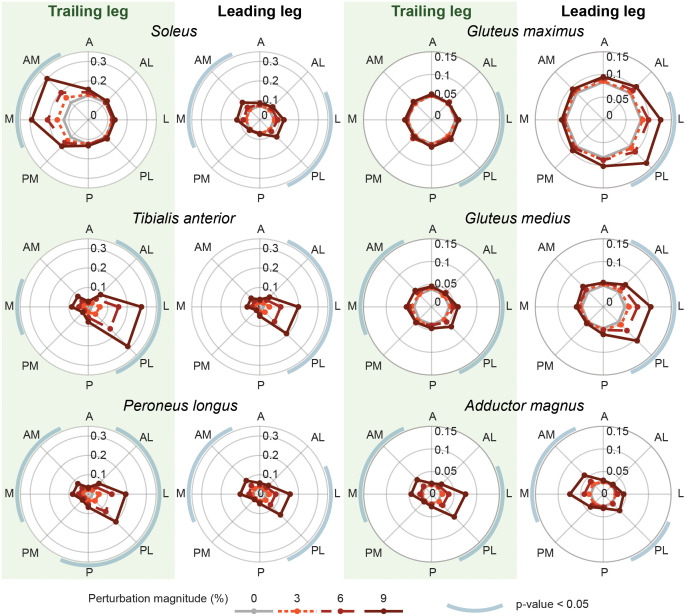
Normalised muscle activity. Muscle activations over the first 1.5 s after the perturbations given in the different directions. The results are presented for the trailing (= left) leg and leading (= right) leg. The line colour and style indicate the perturbation magnitudes. The blue circular arcs around the polar plots indicate whether there is a significant effect of the perturbation on the outcome measure with p < 0.05. All results are based on the averages across all participants.

Due to the asymmetrical position of the feet in staggered stance, we observed different muscle responses in the trailing and leading leg to the same perturbations, Fig 4. The muscles acting around the ankle, the mSOL, mTA and mPL, exhibited a stronger response in the more loaded trailing leg compared to the leading leg. Conversely, the muscles around the hip, the mGMa and mGMe of the leading leg showed more prominent activations compared to those of the trailing leg. Overall, the upper and lower leg muscles of both legs showed a minimal response to perturbations in the A and P direction, compared to other directions. Only the mPL of the trailing leg showed a small, barely significant response to P perturbations (0.006 /%BW, p = 0.049). The perturbations applied in the directions perpendicular to the line between both feet elicited the largest reactions in all measured muscles (up till 0.030 /%BW). For both legs the mPL, inducing plantarflexion and eversion and the mTA inducing dorsiflexion of the ankles, showed significant activations after perturbations in these directions, with the most prominent response seen after L and PL perturbations. The mSOL, inducing plantarflexion, exhibited a larger difference between the trailing and leading leg. The trailing leg mSOL had the largest response to predominantly M and AM perturbations (0.017 and 0.018 /%BW, p < 0.001).

For each leg, the mGMe, a hip abductor, and mGMa, a hip extensor, showed similar reactions for the same perturbation directions. The gluteus muscles of the leading leg showed large significant activation patterns after perturbations in the L and PL directions (0.004 till 0.007 /%BW, always p < 0.001). In comparison, the gluteus muscles of the trailing leg showed smaller responses for these directions (0.001 till 0.002 /%BW, always p < 0.019). Hip adductor mAM of both legs showed a similar reaction to the same perturbations, with the strongest significant activations after perturbations perpendicular to the line between both feet (0.002 till 0.005 /%BW, always p < 0.021), except for the mAM of the leading leg after L perturbations.

### Joint moment response

**Fig 5:**
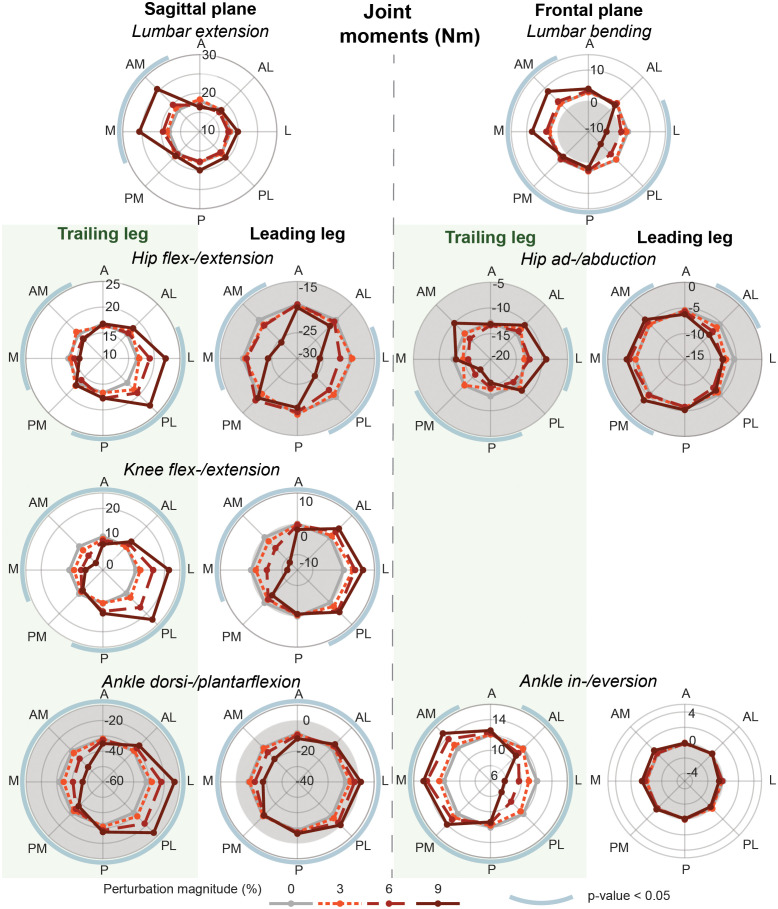
Joint moments. Joint moments of the lumbar, hip, knee and ankle joint in the frontal and sagittal plane in Nm. The mean value over the first 1.5 s, is shown for each perturbation magnitude and direction and the baseline during standing in the staggered stance posture. The line colour and style indicate the perturbation magnitudes. The blue circular arcs around the polar plots indicate whether there is a significant effect of the perturbation on the outcome measure with p < 0.05. The gray background indicates a negative value. All results are based on the averages across all participants.

The strongest joint contributions were observed after perturbations perpendicular to the line between both feet, Fig 5. The lumbar joint mainly contributed in the frontal plane by creating a significant bending moment (-0.78 till 0.59 Nm/%BW, p < 0.050 except for A and AL perturbations), while hardly any significant contributions were shown in the sagittal plane by lumbar extension, except after M and AM perturbations (0.73 and 0.77 Nm/%BW, p < 0.001).

In the frontal plane the strongest joint contributions came from the loaded trailing leg. The hip ab- and adduction moments showed opposite responses in the in- and decrease of the abduction moment for the trailing and leading leg, contributing to bringing the upper body back to the starting position in ML direction. This was done together with an in- or decrease of the ankle inversion of the trailing foot after M and AM or L and PL perturbations respectively. Meanwhile there was no significant contribution to the response by the ankle in- and eversion of the leading leg.

In the sagittal plane the ankle, knee and hip joints of both the trailing and leading leg significantly contributed to the recovery after various perturbation directions. Perturbations perpendicular to the line between both feet strongly increased the hip extension moment of the leading leg (-0.51 till -0.66 Nm/%BW, p < 0.002). For the trailing leg a strong increase of the hip flexion moment was seen after perturbations in L, PL and P direction. Symmetric responses in the change of the flexion/extension and plantar/dorsiflexion moments were seen in the knees and ankles respectively of both the trailing and leading leg. Especially after perturbations perpendicular to the line between both feet a strong increase (after M and AM perturbations) and reduction (after L and PL perturbations) of the ankle plantar flexion moment were presented (-1.73 till 2.33 Nm/%BW, p < 0.001).

## Discussion

This study aimed to establish how activations of the hip, knee and ankle joints in multiple directions contribute and co-operate in balance recovery after pelvis perturbations in various directions while standing in a staggered stance. As reported before by others, the effect of the perturbations on the maintenance of balance was clearly influenced by the dimensions of the BoS [6, 8, 11]. While standing in a staggered stance this made the individuals more sensitive to perturbations given in the ML and diagonal directions, perpendicular to the line between both feet. This higher sensitivity was reflected in a larger number of steps that needed to be taken, a longer time to the point of return, larger deviations of the CoM and stronger responses on muscle and joint level, compared to perturbations approaching collinearity with the line between the feet.

After M or L perturbations, the CoM did not only deviate in ML direction, but also a significant AP motion was present. Remarkably, instead of moving the CoM away from the edges of the BoS, the CoM was brought more forward after the M perturbations and backward after the L perturbations, bringing the CoM closer to the BoS boundaries. Besides the fact that this appears to be counter-intuitive, since we would like to keep our CoM with a margin within the BoS, it was also contrary to what initially could be expected based on the presented ankle plantar-/dorsiflexion moments and contributing muscle activity. However, an explanation for this resulting CoM motion could be that it does assist in bringing the weight distribution back to the original situation [29]. After the M perturbations the ankle response resembled the ankle plantarflexion moment modulation during gait reported by Kim et al. [30]. Based on simulations they showed how the ankle plantarflexion moment can contribute to ML balance during walking [30]. The combination of hip and knee flexion and extension moments contributed to achieving the presented CoM motion, which seems opposite to the findings of Winter et al. [6], during unperturbed staggered stance. They reported a counteraction between the ankle and hip as well, however the other way around, such that the ankle had to compensate for an inappropriate hip contribution.

Another remarkable finding after perturbations in L, PL and AM direction are the unsmooth trajectories of the CoP, Fig 3. While checking the individual repetitions, this effect was present as well, together with a larger variety in the shapes of the CoP trajectories. The reason these perturbations resulted in these unsmooth trajectories and larger variety, could be because the total CoP position is largely dependent on the loading and unloading of both legs [31]. If a perturbation brings the CoM close to the edge of the BoS, the participant might be close to the point that a step is needed for balance recovery. Preparations for making a step involve a weight shift towards the future stance leg. However if this is not needed anymore, it could result in a fast alternating weight shift between both feet.

In general, perturbations collinear with the line between both feet provoked smaller responses on joint level, compared to those perpendicular to the line between both feet. At the same time the excursion of the CoP was large, keeping the CoM deviations small. Especially responses in the frontal plane were small and not always significant after the perturbations collinear with the line between both feet, suggesting a weaker coupling between the sagittal and frontal plane compared to the perturbations perpendicular to the line between both feet. O’Connor et al. [8] extensively reported sensitivities after visual ML and AP perturbations during walking, normal stance and tandem stance. Our findings, while standing in a posture in between normal and tandem stance, revealed sensitivities similar to those reported by O’Connor et al. during walking and tandem stance [8]. This is probably because the shape and direction of the BoS during these postures correspond most with those during staggered stance.

All perturbations were only applied with the right foot leading. Recommendations for future research would be to investigate the balance response in a staggered stance with the left leg leading as well, since the balance response of an individual could be influenced by the position of their dominant foot [10, 31]. Also, the participants were asked to cross their arms over their abdomen to prevent them from using arm swing as balance strategy. Swinging the arms and grabbing the adjacent rails as a reflex could assist in a natural balance response. This may have led to a psychological influence on the necessity of stepping. Besides, with the used setup it was also not possible to leave the arms along the body because of the rod connection with the motor on the side.

The obtained results give insights in balance recovery during staggered stance, a posture becoming important in situations for example while standing in public transport, while walking very slowly, or while wearing a lower-limb exoskeleton due to a movement disorder making walking less dynamic. However, we should keep in mind that the results obtained during this static posture could not directly be translated to balance recovery during gait. The results of this study provide fundamental insights into balance properties and abilities in this posture. This could facilitate improvement of future designs of exoskeleton controllers that assist paraplegics during walking.

## Conclusion

While standing in staggered stance, pelvis perturbations perpendicular to the line between both feet required strong joint responses in order to maintain balance. Reactions in both the frontal and sagittal planes contributed to the recovery of these perturbations. In contrast, perturbations collinear with the line between both feet revealed smaller responses and less coupling between responses in the sagittal and frontal plane.

## Supporting information

S1 File Time series results. File containing the time series over the first 1.5 s after the perturbations for the various outcome measures after the different perturbation directions and magnitudes.
(PDF)

S2 File Statistical results. File containing tables with the results of all statistical tests.
(PDF)
