# Supplementary material for: Pelvis perturbations in various directions while standing in staggered stance elicit concurrent responses in both the sagittal and frontal plane
Source: PLoS One. 2023 Apr 12;18(4):e0272245. doi: 10.1371/journal.pone.0272245 (PMC10096271; doi:10.1371/journal.pone.0272245)
Supplement: S1 File — File containing the time series over the first 1.5 s after the perturbations for the various outcome measures after the different perturbation directions and magnitudes. (PDF) [file pone.0272245.s001.pdf]

S1. Fig. Times series results

Supplementary material to:

Pelvis perturbations in various directions while standing in staggered stance elicit concurrent responses in both the sagittal and frontal plane

Michelle van Mierlo[1¶*], Jean A. Ormiston[1,2¶], Mark Vlutters[1], Edwin H.F. van Asseldonk[1], Herman van der Kooij[1,3],

1 Department of Biomechanical Engineering, University of Twente, Enschede, The Netherlands
2 Department of Research, Sint Maartenskliniek, Nijmegen, The Netherlands
3 Department of Biomechanical Engineering, Delft University of Technology, Delft, The Netherlands

¶These authors contributed equally to this work.
* corresponding author: m.vanmierlo@utwente.nl

Introduction

This document presents time series over the first 1.5 s from the start of a perturbation for the various outcome measures: centre of mass (CoM) and centre of pressure (CoP) position, EMG activities and joint moments. The results present the average response over all repetitions and participants. For the full methods on how these measures were obtained we would like to refer to the main paper. The figure in the middle indicates the perturbation directions for the graphs presented around it. The results are ordered per outcome measure:

- CoM & CoP 2
- M. Soleus 3
- M. Tibialis anterior 3
- M. Peroneus longus 4
- M. Gluteus maximus 4
- M. Gluteus medius 5
- M. Adductor magnus 5
- Lumbar joint 6
- Hip joint 7
- Knee joint 8
- Ankle joint 9

CoM and CoP position

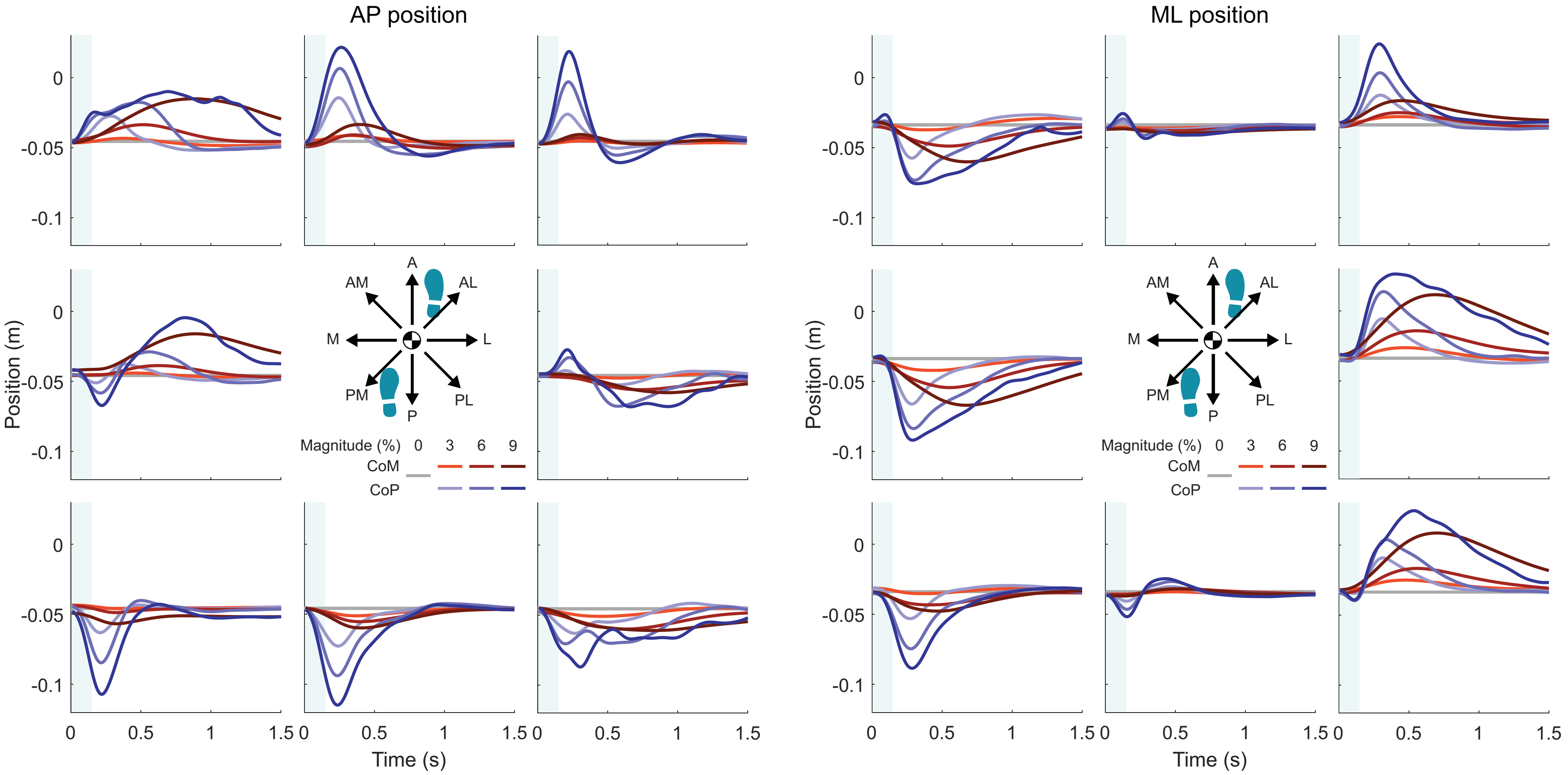

Muscle activity

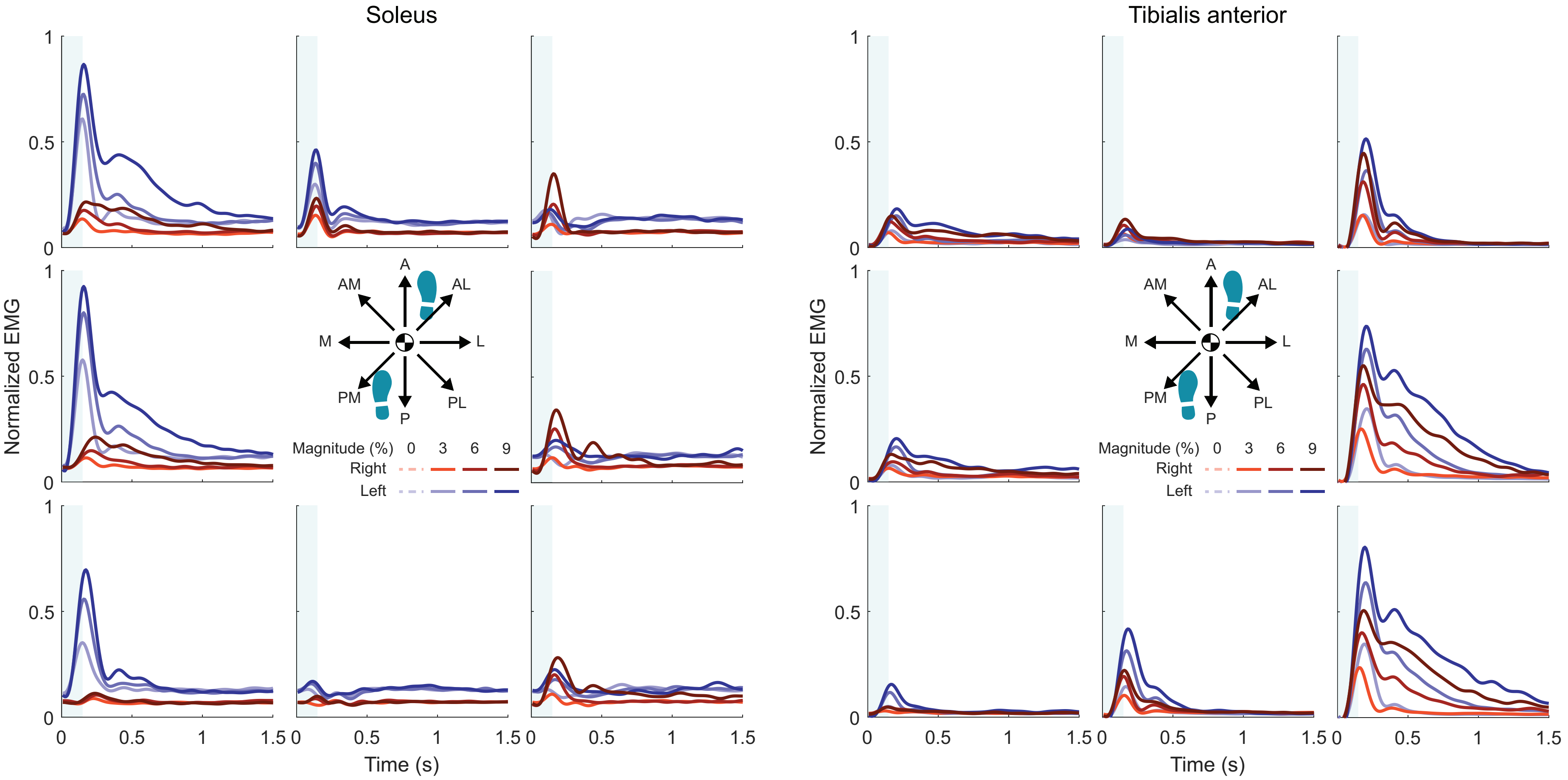

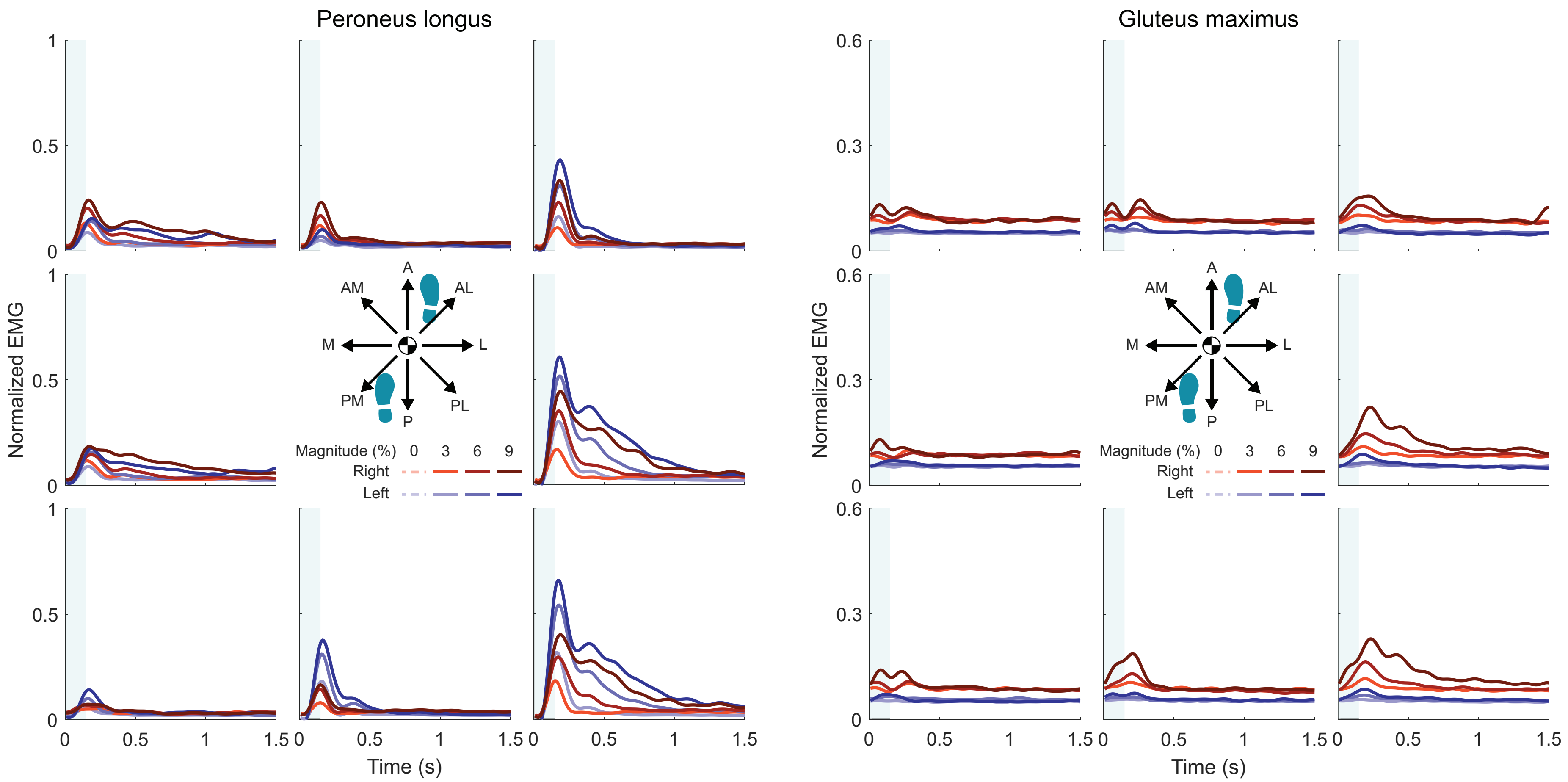
Peroneus longus
Gluteus maximus
Normalized EMG
Time (s)
A
AM
AL
M
L
PM
P
PL
Magnitude (%) 0 3 6 9
Right
Left

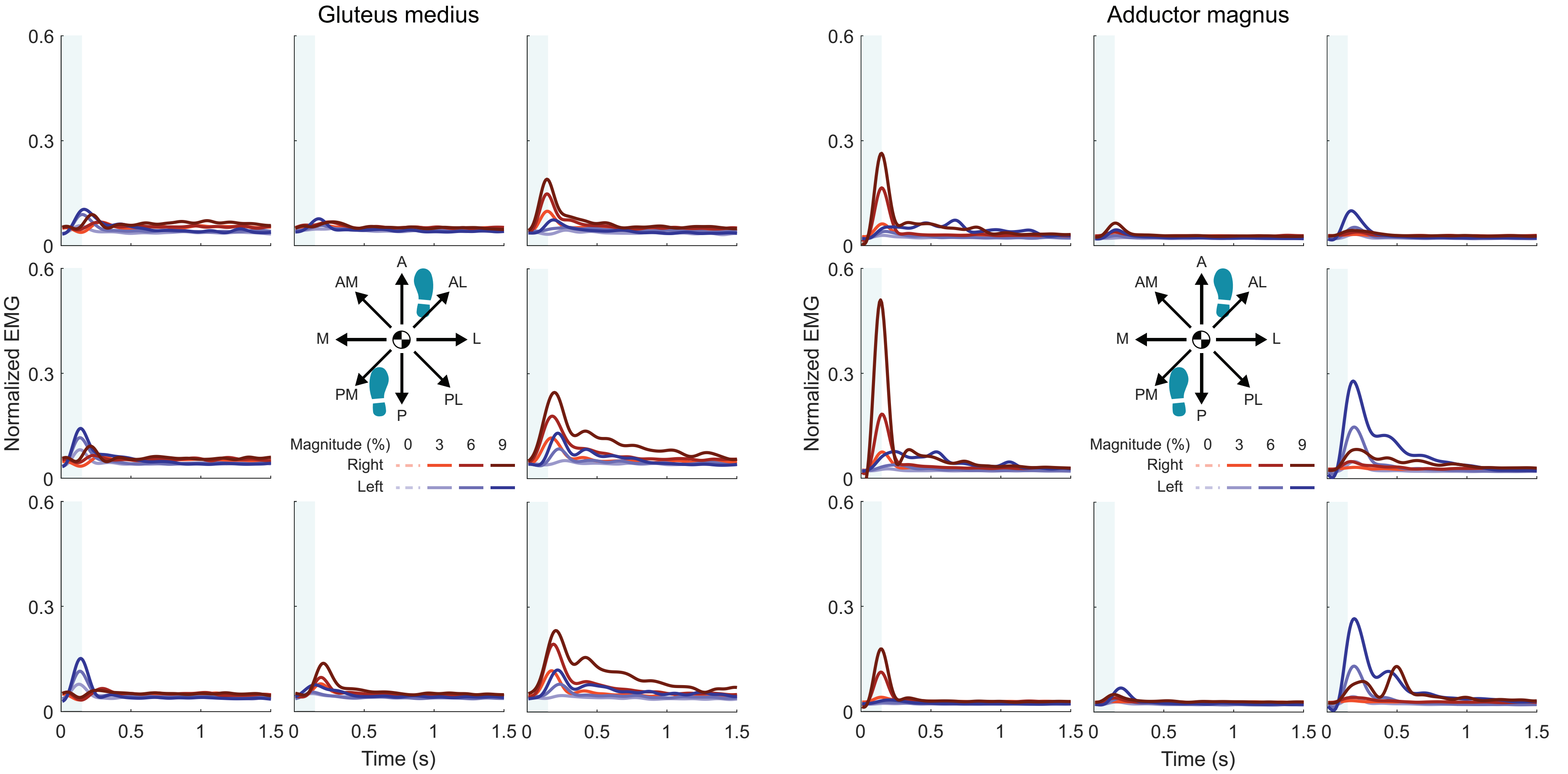

Gluteus medius
Adductor magnus
Normalized EMG
Time (s)
0.6
0.3
0
0.5
1
1.5
A
AM
AL
M
L
PM
PL
P
Magnitude (%) 0 3 6 9
Right
Left

Joint moments

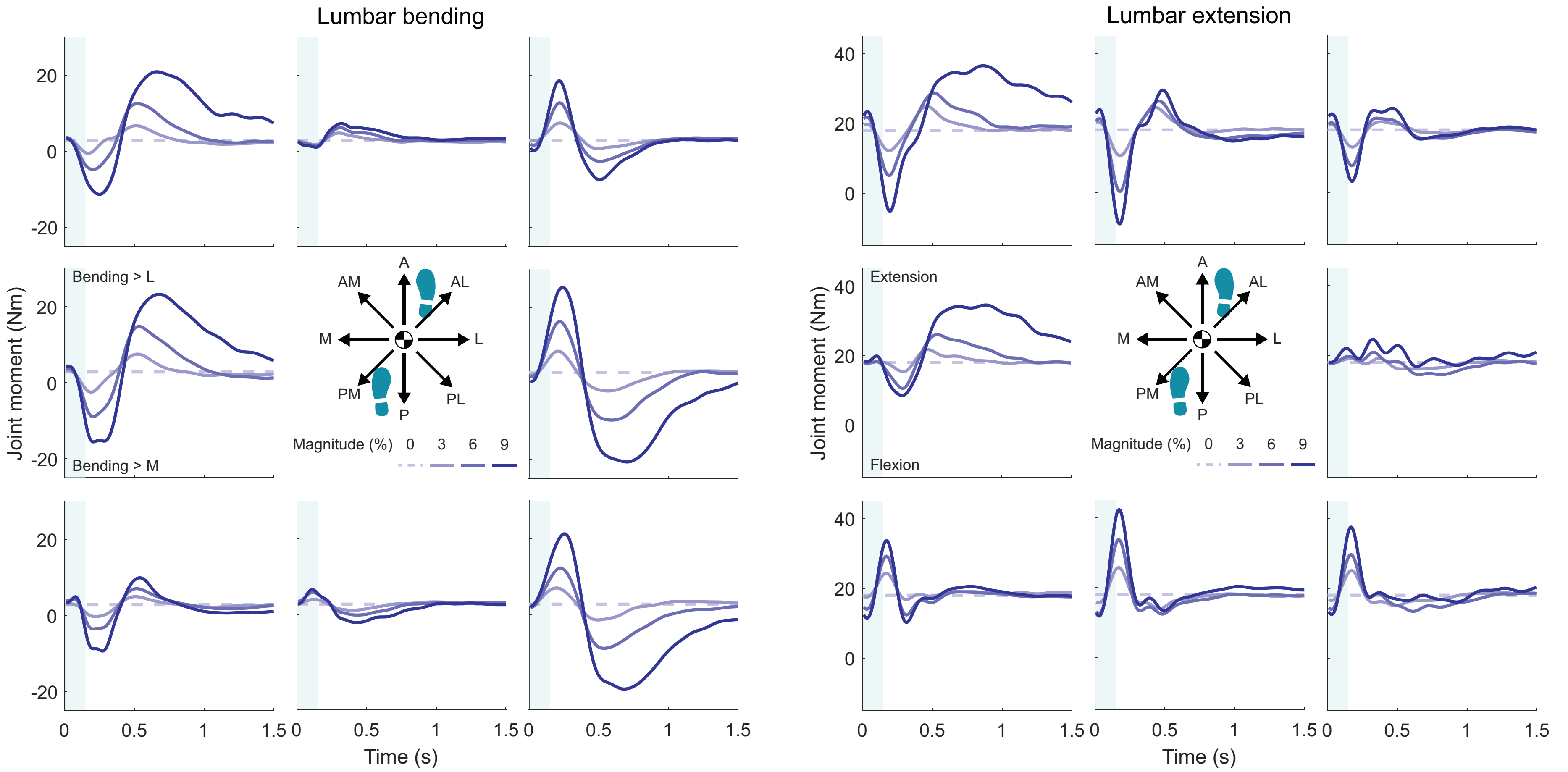

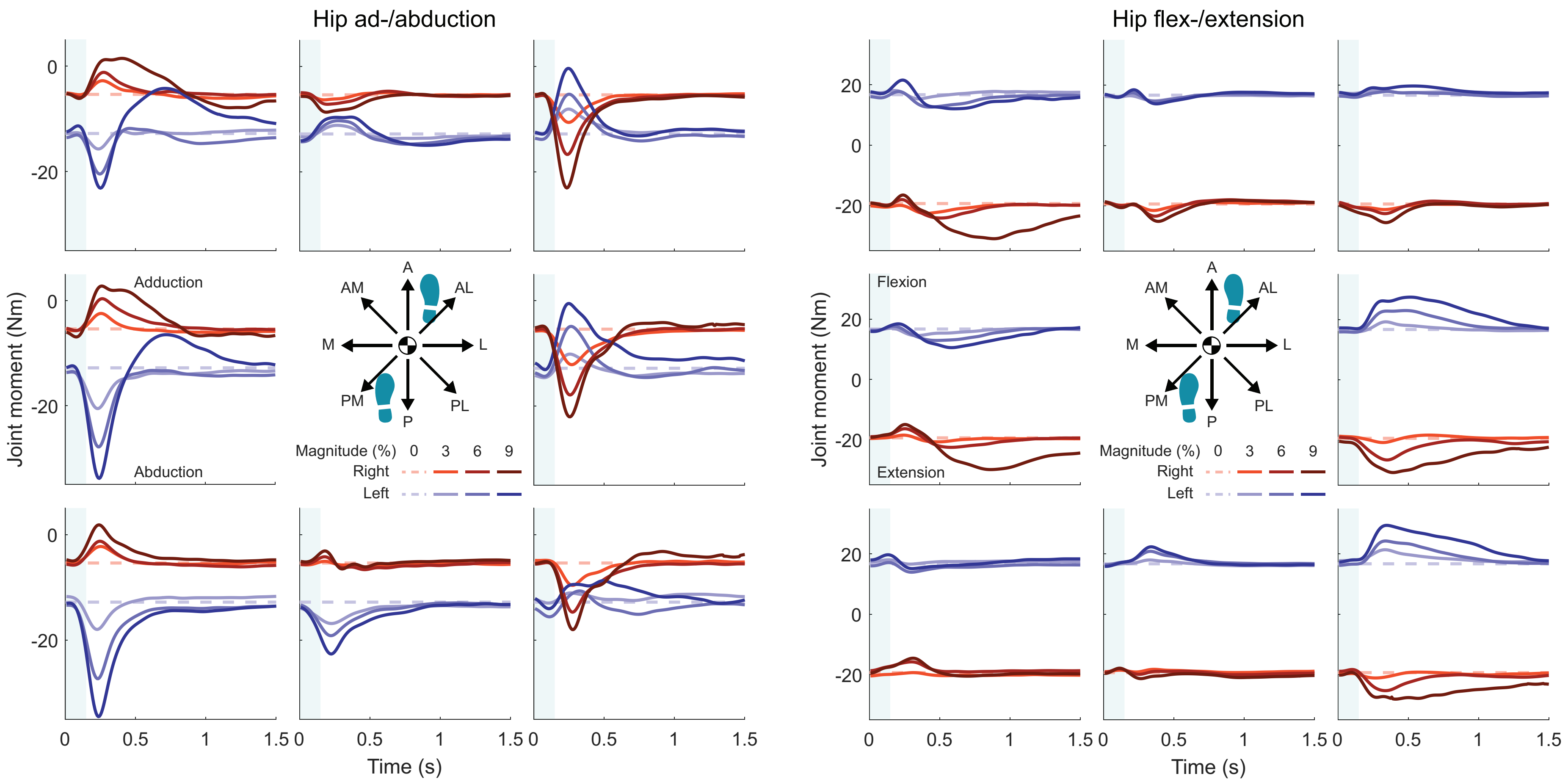
Hip ad-/abduction
Hip flex-/extension
Joint moment (Nm)
Adduction
Abduction
Flexion
Extension
A
AM
AL
M
L
PM
PL
P
Magnitude (%) 0 3 6 9
Right
Left
Time (s)

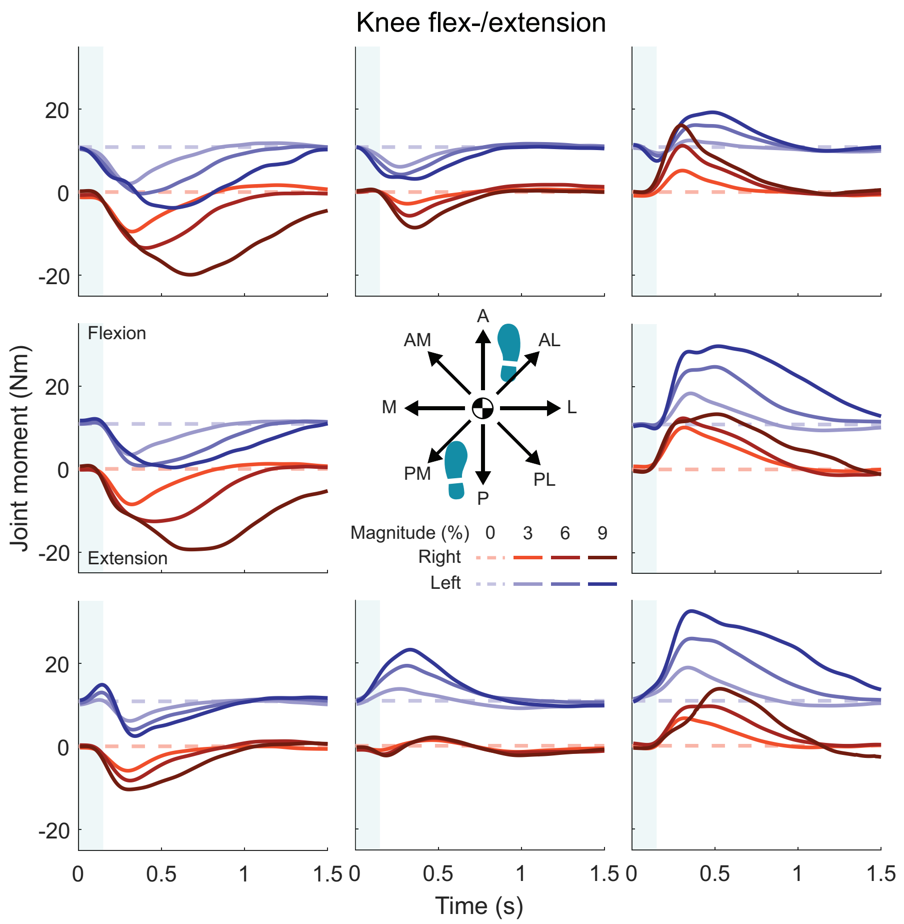

Knee flex-/extension
Joint moment (Nm)
Flexion
Extension
20
0
-20
A
AM
AL
M
L
PM
PL
P
Magnitude (%) 0 3 6 9
Right
Left
0
0.5
1
1.5
Time (s)

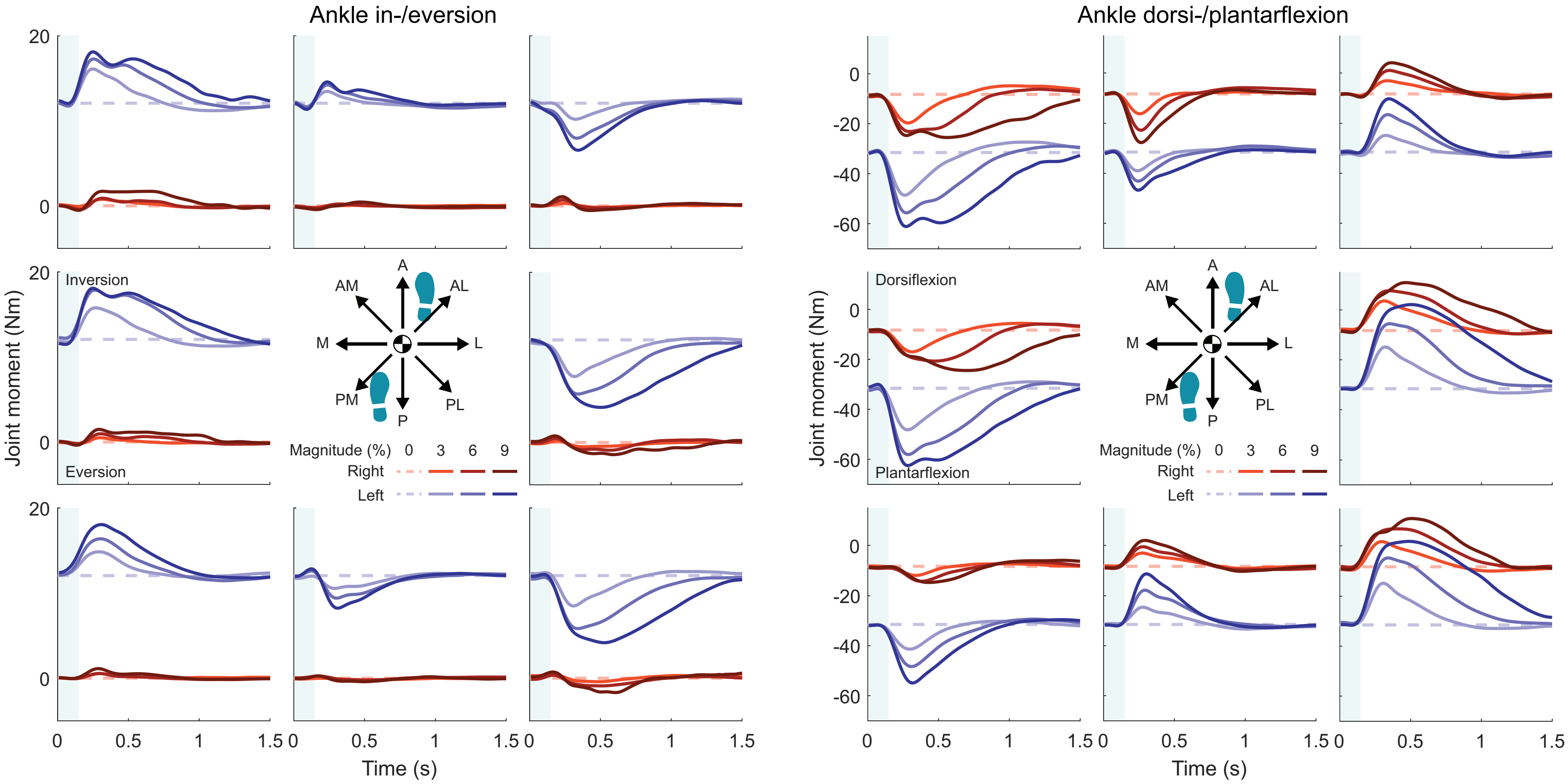
Ankle in-/eversion
Ankle dorsi-/plantarflexion
Joint moment (Nm)
Inversion
Eversion
Dorsiflexion
Plantarflexion
A
AM
AL
M
L
PM
PL
P
Magnitude (%) 0 3 6 9
Right
Left
Time (s)
20
0
-20
-40
-60
0.5
1
1.5
