# Supplementary material for: Pelvis perturbations in various directions while standing in staggered stance elicit concurrent responses in both the sagittal and frontal plane
Source: PLoS One. 2023 Apr 12;18(4):e0272245. doi: 10.1371/journal.pone.0272245 (PMC10096271; doi:10.1371/journal.pone.0272245)
Supplement: S2 File — File containing tables with the results of all statistical tests. (PDF) [file pone.0272245.s002.pdf]

S1. Table. Statistical results

Supplementary material to:

Pelvis perturbations in various directions while standing in staggered stance elicit concurrent responses in both the sagittal and frontal plane

Michelle van Mierlo[1¶*], Jean A. Ormiston[1,2¶], Mark Vlutters[1], Edwin H.F. van Asseldonk[1], Herman van der Kooij[1,3],

1 Department of Biomechanical Engineering, University of Twente, Enschede, The Netherlands
2 Department of Research, Sint Maartenskliniek, Nijmegen, The Netherlands
3 Department of Biomechanical Engineering, Delft University of Technology, Delft, The Netherlands

¶These authors contributed equally to this work.
* corresponding author: m.vanmierlo@utwente.nl

Introduction

This document contains extensive tables presenting the results of the statistical tests for the different outcome measures: the centre of mass (CoM) position, centre of pressure (CoP) position, EMG activities and joint moments. For the full methods on how the linear mixed model analysis was performed we would like to refer to the main paper. The results are ordered per outcome measure.

- CoM 2
- CoP 3
- M. Soleus 4
- M. Tibialis anterior 5
- M. Peroneus longus 6
- M. Gluteus maximus 7
- M. Adductor magnus 8
- M. Gluteus medius 9
- Lumbar joint 10
- Hip adduction 11
- Ankle inversion 12
- Hip flexion 13
- Knee flexion 14
- Ankle dorsiflexion 15

Centre of mass

	AP CoM (cm)				ML CoM (cm)			
**Fixed effects**	**Parameter**	**df**	**t**	**p-value**	**Parameter**	**df**	**t**	**p-value**
Intercept	-0.14	223.2	-0.53	0.595	0.13	286.5	0.97	0.334
Magnitude	0.16	39.0	2.59	0.014	-0.02	98.9	-0.80	0.423
Direction AL	0.14	301.0	0.38	0.703	-0.18	301.1	-1.00	0.320
Direction L	0.37	301.0	1.01	0.313	-0.55	301.2	-2.99	0.003
Direction PL	0.44	301.0	1.17	0.243	-0.56	301.1	-3.03	0.003
Direction P	0.24	301.0	0.64	0.520	-0.17	301.1	-0.91	0.362
Direction PM	0.12	301.0	0.33	0.742	-0.04	301.1	-0.20	0.845
Direction M	-0.41	301.0	-1.11	0.269	0.26	301.1	1.42	0.155
Direction AM	-0.50	301.0	-1.34	0.182	0.34	301.1	1.86	0.064
Magnitude:Direction AL	-0.11	301.2	-1.58	0.115	0.20	301.6	6.00	<0.001
Magnitude:Direction L	-0.36	301.3	-5.38	<0.001	0.54	301.7	16.22	<0.001
Magnitude:Direction PL	-0.43	301.2	-6.38	<0.001	0.50	301.6	15.20	<0.001
Magnitude:Direction P	-0.31	301.0	-4.77	<0.001	0.07	301.1	2.11	0.035
Magnitude:Direction PM	-0.24	301.2	-3.61	<0.001	-0.12	301.6	-3.65	<0.001
Magnitude:Direction M	0.14	301.0	2.20	0.029	-0.39	301.1	-11.82	<0.001
Magnitude:Direction AM	0.24	301.2	3.60	<0.001	-0.32	301.6	-9.78	<0.001
**Random effects**								
Std intercept	0.002				<0.001			
Std PertMag	0.001				<0.001			
Correlation	-1.00				-1.00			
**Model fit**								
R^2-marginal	0.46				0.90			
R^2-conditional	0.59				0.91			

Centre of pressure

	AP CoP (cm)				ML CoP (cm)			
**Fixed effects**	**Parameter**	**df**	**t**	**p-value**	**Parameter**	**df**	**t**	**p-value**
Intercept	0.31	269.2	0.72	0.472	0.11	301.4	0.77	0.443
Magnitude	0.84	71.6	9.30	<0.001	-0.03	267.9	-1.14	0.256
Direction AL	-0.36	301.0	-0.59	0.557	-0.06	301.4	-0.30	0.767
Direction L	0.25	301.0	0.40	0.688	0.08	301.5	0.40	0.691
Direction PL	0.31	301.0	0.50	0.619	-0.27	301.4	-1.29	0.197
Direction P	-0.43	301.0	-0.70	0.488	-0.15	301.4	-0.71	0.478
Direction PM	-0.30	301.0	-0.49	0.623	-0.29	301.4	-1.39	0.166
Direction M	-0.84	301.0	-1.37	0.172	-0.56	301.4	-2.73	0.007
Direction AM	-0.62	301.0	-1.01	0.315	-0.39	301.4	-1.86	0.064
Magnitude:Direction AL	-0.08	301.4	-0.74	0.461	0.68	302.2	18.17	<0.001
Magnitude:Direction L	-1.45	301.4	-13.11	<0.001	0.89	302.4	23.93	<0.001
Magnitude:Direction PL	-1.83	301.4	-16.54	<0.001	0.87	302.2	23.26	<0.001
Magnitude:Direction P	-1.63	301.0	-14.94	<0.001	0.06	301.4	1.64	0.101
Magnitude:Direction PM	-1.53	301.4	-13.83	<0.001	-0.59	302.2	-15.90	<0.001
Magnitude:Direction M	-0.37	301.0	-3.43	0.001	-0.66	301.4	-17.86	<0.001
Magnitude:Direction AM	-0.04	301.4	-0.40	0.689	-0.54	302.2	-14.51	<0.001
**Random effects**								
Std intercept	0.002				<0.001			
Std PertMag	0.001				<0.001			
Correlation	-1.00				-			
**Model fit**								
R^2-marginal	0.84				0.98			
R^2-conditional	0.86				0.98			

Soleus

	Soleus trailing leg				Soleus leading leg			
**Fixed effects**	**Parameter**	**df**	**t**	**p-value**	**Parameter**	**df**	**t**	**p-value**
Intercept	0.123	16.5	8.38	<0.001	0.071	14.1	7.48	<0.001
Magnitude	0.003	26.9	1.78	0.086	0.001	37.6	1.53	0.135
Direction AL	0.003	291.1	0.31	0.757	-0.002	291.2	-0.34	0.735
Direction L	0.001	291.1	0.06	0.953	-0.005	291.2	-0.87	0.387
Direction PL	<|0.001|	291.1	0.04	0.972	-0.006	291.2	-1.06	0.291
Direction P	-0.001	291.0	-0.08	0.936	-0.001	291.1	-0.13	0.895
Direction PM	<|0.001|	291.1	0.01	0.990	<|0.001|	291.2	-0.02	0.982
Direction M	-0.010	291.0	-0.92	0.359	-0.008	291.1	-1.33	0.185
Direction AM	-0.011	291.1	-1.03	0.306	-0.005	291.2	-0.88	0.380
Magnitude:Direction AL	-0.003	291.2	-1.81	0.071	0.001	291.4	1.01	0.313
Magnitude:Direction L	-0.003	291.2	-1.65	0.100	0.004	291.4	3.93	<0.001
Magnitude:Direction PL	-0.003	291.2	-1.50	0.134	0.004	291.4	3.52	<0.001
Magnitude:Direction P	-0.002	291.0	-1.31	0.190	-0.001	291.1	-1.25	0.212
Magnitude:Direction PM	0.003	291.2	1.81	0.071	-0.001	291.4	-1.13	0.261
Magnitude:Direction M	0.015	291.0	7.95	<0.001	0.004	291.1	3.50	0.001
Magnitude:Direction AM	0.014	291.2	7.46	<0.001	0.004	291.4	3.95	<0.001
**Random effects**								
Std intercept	0.040				0.027			
Std PertMag	0.005				0.002			
Correlation	-0.10				-0.54			
**Model fit**								
R^2-marginal	0.38				0.21			
R^2-conditional	0.83				0.78			

Tibialis anterior

	Tibialis anterior trailing leg				Tibialis anterior leading leg			
**Fixed effects**	**Parameter**	**df**	**t**	**p-value**	**Parameter**	**df**	**t**	**p-value**
Intercept	0.015	301.1	1.36	0.173	0.020	112.7	2.85	0.005
Magnitude	0.001	36.7	0.49	0.624	0.002	37.1	1.31	0.198
Direction AL	-0.005	301.1	-0.31	0.754	-0.004	300.9	-0.43	0.669
Direction L	-0.020	301.1	-1.25	0.212	-0.016	300.9	-1.76	0.079
Direction PL	-0.023	301.1	-1.47	0.143	-0.018	300.9	-1.95	0.052
Direction P	-0.002	301.1	-0.15	0.880	<|0.001|	300.9	-0.04	0.964
Direction PM	-0.001	301.1	-0.04	0.967	<|0.001|	300.9	-0.02	0.984
Direction M	-0.005	301.1	-0.33	0.741	-0.003	300.9	-0.33	0.744
Direction AM	-0.004	301.1	-0.25	0.803	-0.003	300.9	-0.29	0.770
Magnitude:Direction AL	0.007	301.2	2.43	0.016	0.004	300.9	2.68	0.008
Magnitude:Direction L	0.028	301.2	9.62	<0.001	0.018	301.0	11.14	<0.001
Magnitude:Direction PL	0.029	301.2	10.16	<0.001	0.018	300.9	11.00	<0.001
Magnitude:Direction P	0.006	301.1	1.95	0.052	0.001	300.9	0.64	0.522
Magnitude:Direction PM	0.001	301.2	0.48	0.635	-0.001	300.9	-0.52	0.602
Magnitude:Direction M	0.006	301.1	2.21	0.028	0.003	300.9	1.72	0.087
Magnitude:Direction AM	0.005	301.2	1.83	0.068	0.003	300.9	1.96	0.051
**Random effects**								
Std intercept	<0.001				0.009			
Std PertMag	0.006				0.003			
Correlation	-				1.00			
**Model fit**								
R^2-marginal	0.59				0.61			
R^2-conditional	0.75				0.81			

Peroneus longus

	Peroneus longus trailing leg				Peroneus longus leading leg			
**Fixed effects**	**Parameter**	**df**	**t**	**p-value**	**Parameter**	**df**	**t**	**p-value**
Intercept	0.020	159.1	2.53	0.012	0.036	41.8	4.16	<0.001
Magnitude	0.002	27.6	0.75	0.460	0.002	50.5	1.55	0.127
Direction AL	-0.003	291.3	-0.31	0.756	-0.005	290.9	-0.51	0.612
Direction L	-0.010	291.1	-0.97	0.335	-0.015	290.9	-1.55	0.122
Direction PL	-0.013	291.3	-1.20	0.231	-0.014	290.9	-1.52	0.129
Direction P	-0.001	291.1	-0.09	0.930	-0.002	290.8	-0.23	0.821
Direction PM	-0.001	291.3	-0.08	0.936	-0.001	290.9	-0.11	0.910
Direction M	-0.003	291.1	-0.32	0.752	-0.010	290.8	-1.08	0.283
Direction AM	-0.004	291.3	-0.40	0.693	-0.007	290.9	-0.77	0.440
Magnitude:Direction AL	0.005	291.4	2.57	0.011	0.001	291.4	0.69	0.491
Magnitude:Direction L	0.018	291.3	9.22	<0.001	0.011	291.4	6.36	<0.001
Magnitude:Direction PL	0.019	291.4	9.83	<0.001	0.010	291.4	5.97	<0.001
Magnitude:Direction P	0.004	291.1	1.98	0.049	-0.001	290.8	-0.38	0.705
Magnitude:Direction PM	0.001	291.4	0.52	0.606	-0.002	291.4	-1.02	0.309
Magnitude:Direction M	0.005	291.1	2.61	0.010	0.005	290.8	2.91	0.004
Magnitude:Direction AM	0.005	291.4	2.36	0.019	0.005	291.4	2.77	0.006
**Random effects**								
Std intercept	0.007				0.017			
Std PertMag	0.005				0.003			
Correlation	-0.10				-0.86			
**Model fit**								
R^2-marginal	0.57				0.51			
R^2-conditional	0.76				0.61			

Gluteus maximus

	Gluteus maximus trailing leg				Gluteus maximus leading leg			
**Fixed effects**	**Parameter**	**df**	**t**	**p-value**	**Parameter**	**df**	**t**	**p-value**
Intercept	0.054	10.2	6.21	<0.001	0.083	11.0	7.17	<0.001
Magnitude	<|0.001|	109.1	1.57	0.120	0.001	19.8	1.69	0.107
Direction AL	<|0.001|	291.0	0.11	0.912	-0.001	291.1	-0.21	0.835
Direction L	-0.001	291.0	-0.64	0.522	-0.006	291.1	-1.49	0.136
Direction PL	-0.001	291.0	-0.67	0.502	-0.007	291.1	-1.76	0.079
Direction P	<|0.001|	290.9	-0.20	0.844	<|0.001|	291.0	0.03	0.972
Direction PM	0.001	291.0	0.40	0.692	0.001	291.1	0.17	0.866
Direction M	<|0.001|	290.9	-0.25	0.799	0.001	291.0	0.21	0.835
Direction AM	-0.001	291.0	-0.94	0.348	0.002	291.1	0.42	0.676
Magnitude:Direction AL	<|0.001|	291.8	-0.23	0.819	0.001	291.2	0.89	0.373
Magnitude:Direction L	0.001	291.7	2.38	0.018	0.003	291.2	4.91	<0.001
Magnitude:Direction PL	0.001	291.8	2.63	0.009	0.004	291.2	5.98	<0.001
Magnitude:Direction P	<|0.001|	290.9	1.09	0.275	<|0.001|	291.0	0.64	0.524
Magnitude:Direction PM	<|0.001|	291.8	0.20	0.844	<|0.001|	291.2	-0.43	0.669
Magnitude:Direction M	<|0.001|	290.9	0.71	0.479	-0.001	291.0	-0.76	0.447
Magnitude:Direction AM	<|0.001|	291.8	1.32	0.186	<|0.001|	291.2	-0.33	0.741
**Random effects**								
Std intercept	0.027				0.036			
Std PertMag	<0.001				0.002			
Correlation	0.80				0.02			
**Model fit**								
R^2-marginal	0.01				0.07			
R^2-conditional	0.98				0.94			

Adductor magnus

	Adductor magnus trailing leg				Adductor magnus leading leg			
**Fixed effects**	**Parameter**	**df**	**t**	**p-value**	**Parameter**	**df**	**t**	**p-value**
Intercept	0.021	38.2	5.01	<0.001	0.027	14.1	3.56	0.003
Magnitude	$<\lvert 0.001 \rvert$	58.8	0.51	0.614	$<\lvert 0.001 \rvert$	82.6	0.63	0.528
Direction AL	-0.001	291.3	-0.21	0.830	0.001	291.0	0.24	0.812
Direction L	-0.007	291.2	-1.69	0.093	-0.002	291.0	-0.44	0.659
Direction PL	-0.007	291.3	-1.52	0.130	-0.003	291.0	-0.67	0.504
Direction P	$<\lvert 0.001 \rvert$	291.2	-0.01	0.994	0.001	290.9	0.18	0.855
Direction PM	0.001	291.3	0.13	0.900	0.001	291.0	0.13	0.900
Direction M	-0.004	291.2	-0.81	0.420	-0.005	290.9	-1.08	0.283
Direction AM	-0.002	291.3	-0.56	0.577	-0.001	291.0	-0.30	0.765
Magnitude:Direction AL	0.001	291.8	0.84	0.401	$<\lvert 0.001 \rvert$	291.5	-0.22	0.824
Magnitude:Direction L	0.005	291.7	6.84	<0.001	0.001	291.4	1.70	0.091
Magnitude:Direction PL	0.005	291.8	6.13	<0.001	0.002	291.5	2.33	0.021
Magnitude:Direction P	$<\lvert 0.001 \rvert$	291.2	0.42	0.678	$<\lvert 0.001 \rvert$	290.9	-0.11	0.915
Magnitude:Direction PM	$<\lvert 0.001 \rvert$	291.8	-0.21	0.831	0.001	291.5	1.05	0.292
Magnitude:Direction M	0.002	291.2	2.81	0.005	0.004	290.9	5.56	<0.001
Magnitude:Direction AM	0.002	291.8	2.42	0.016	0.003	291.5	3.39	0.001
**Random effects**								
Std intercept	0.009				0.021			
Std PertMag	0.001				0.001			
Correlation	0.69				-0.07			
**Model fit**								
R^2-marginal	0.31				0.13			
R^2-conditional	0.70				0.79			

Gluteus medius

<table>
<tr><th></th><th colspan="4">Gluteus medius trailing leg</th><th colspan="4">Gluteus medius leading leg</th></tr>
<tr><th>Fixed effects</th><th>Parameter</th><th>df</th><th>t</th><th>p-value</th><th>Parameter</th><th>df</th><th>t</th><th>p-value</th></tr>
<tr><td>Intercept</td><td>0.042</td><td>10.9</td><td>5.01</td><td><0.001</td><td>0.049</td><td>11.0</td><td>4.87</td><td><0.001</td></tr>
<tr><td>Magnitude</td><td><|0.001|</td><td>49.9</td><td>0.98</td><td>0.334</td><td>0.001</td><td>65.9</td><td>1.22</td><td>0.228</td></tr>
<tr><td>Direction AL</td><td>-0.001</td><td>290.5</td><td>-0.42</td><td>0.678</td><td>-0.002</td><td>291.2</td><td>-0.57</td><td>0.566</td></tr>
<tr><td>Direction L</td><td>-0.004</td><td>290.5</td><td>-1.40</td><td>0.162</td><td>-0.007</td><td>291.1</td><td>-1.99</td><td>0.048</td></tr>
<tr><td>Direction PL</td><td>-0.004</td><td>290.5</td><td>-1.51</td><td>0.133</td><td>-0.007</td><td>291.2</td><td>-2.12</td><td>0.035</td></tr>
<tr><td>Direction P</td><td>-0.002</td><td>290.4</td><td>-0.80</td><td>0.423</td><td>-0.003</td><td>291.1</td><td>-0.86</td><td>0.392</td></tr>
<tr><td>Direction PM</td><td>0.001</td><td>290.5</td><td>0.19</td><td>0.852</td><td>-0.002</td><td>291.2</td><td>-0.50</td><td>0.616</td></tr>
<tr><td>Direction M</td><td>-0.002</td><td>290.4</td><td>-0.56</td><td>0.573</td><td>-0.001</td><td>291.1</td><td>-0.45</td><td>0.653</td></tr>
<tr><td>Direction AM</td><td>-0.003</td><td>290.5</td><td>-1.11</td><td>0.267</td><td>-0.002</td><td>291.2</td><td>-0.51</td><td>0.610</td></tr>
<tr><td>Magnitude:Direction AL</td><td><|0.001|</td><td>291.1</td><td>0.47</td><td>0.641</td><td>0.002</td><td>291.6</td><td>2.79</td><td>0.006</td></tr>
<tr><td>Magnitude:Direction L</td><td>0.002</td><td>291.0</td><td>4.04</td><td><0.001</td><td>0.006</td><td>291.5</td><td>9.49</td><td><0.001</td></tr>
<tr><td>Magnitude:Direction PL</td><td>0.002</td><td>291.1</td><td>4.54</td><td><0.001</td><td>0.006</td><td>291.6</td><td>9.53</td><td><0.001</td></tr>
<tr><td>Magnitude:Direction P</td><td><|0.001|</td><td>290.4</td><td>0.51</td><td>0.612</td><td>0.001</td><td>291.1</td><td>1.31</td><td>0.190</td></tr>
<tr><td>Magnitude:Direction PM</td><td>0.001</td><td>291.1</td><td>1.62</td><td>0.107</td><td><|0.001|</td><td>291.6</td><td>-0.29</td><td>0.775</td></tr>
<tr><td>Magnitude:Direction M</td><td>0.001</td><td>290.4</td><td>2.50</td><td>0.013</td><td>0.001</td><td>291.1</td><td>1.10</td><td>0.271</td></tr>
<tr><td>Magnitude:Direction AM</td><td>0.001</td><td>291.1</td><td>2.52</td><td>0.012</td><td>0.001</td><td>291.6</td><td>1.63</td><td>0.104</td></tr>
<tr><td>Random effects</td><td></td><td></td><td></td><td></td><td></td><td></td><td></td><td></td></tr>
<tr><td>Std intercept</td><td>0.026</td><td></td><td></td><td></td><td>0.031</td><td></td><td></td><td></td></tr>
<tr><td>Std PertMag</td><td>0.001</td><td></td><td></td><td></td><td>0.001</td><td></td><td></td><td></td></tr>
<tr><td>Correlation</td><td>0.75</td><td></td><td></td><td></td><td>0.32</td><td></td><td></td><td></td></tr>
<tr><td>Model fit</td><td></td><td></td><td></td><td></td><td></td><td></td><td></td><td></td></tr>
<tr><td>R^2-marginal</td><td>0.04</td><td></td><td></td><td></td><td>0.14</td><td></td><td></td><td></td></tr>
<tr><td>R^2-conditional</td><td>0.94</td><td></td><td></td><td></td><td>0.94</td><td></td><td></td><td></td></tr>
</table>

Lumbar joint

	Lumbar bending moment (Nm)				Lumbar flexion moment (Nm)			
**Fixed effects**	**Parameter**	**df**	**t**	**p-value**	**Parameter**	**df**	**t**	**p-value**
Intercept	2.782	23.5	3.74	0.001	17.82	11.5	6.22	<0.001
Magnitude	0.107	229.5	1.27	0.205	-0.14	57.1	-0.85	0.400
Direction AL	0.280	291.6	0.42	0.676	-0.33	291.2	-0.30	0.767
Direction L	0.885	291.6	1.33	0.186	-0.27	291.1	-0.24	0.807
Direction PL	1.161	291.6	1.73	0.084	-0.67	291.2	-0.59	0.555
Direction P	0.361	291.4	0.54	0.588	-0.43	291.1	-0.38	0.702
Direction PM	0.271	291.6	0.40	0.686	0.03	291.2	0.03	0.976
Direction M	-0.964	291.4	-1.45	0.148	-0.76	291.1	-0.67	0.501
Direction AM	-0.919	291.6	-1.37	0.171	-0.83	291.2	-0.73	0.465
Magnitude:Direction AL	-0.178	292.5	-1.48	0.140	0.10	291.5	0.50	0.619
Magnitude:Direction L	-0.823	292.4	-6.86	<0.001	0.26	291.5	1.27	0.204
Magnitude:Direction PL	-0.889	292.5	-7.40	<0.001	0.29	291.5	1.41	0.158
Magnitude:Direction P	-0.234	291.4	-1.97	0.050	0.36	291.1	1.82	0.070
Magnitude:Direction PM	-0.250	292.5	-2.08	0.038	0.20	291.5	1.01	0.314
Magnitude:Direction M	0.462	291.4	3.89	<0.001	0.92	291.1	4.58	<0.001
Magnitude:Direction AM	0.484	292.5	4.02	<0.001	0.87	291.5	4.30	<0.001
**Random effects**								
Std intercept	1.819				8.698			
Std PertMag	0.023				0.304			
Correlation	-0.31				-0.06			
**Model fit**								
R^2-marginal	0.42				0.04			
R^2-conditional	0.71				0.90			

Hip adduction

	Hip adduction moment trailing leg (Nm)				Hip adduction moment leading leg (Nm)			
**Fixed effects**	**Parameter**	**df**	**t**	**p-value**	**Parameter**	**df**	**t**	**p-value**
Intercept	-13.59	11.3	-5.53	<0.001	-5.14	10.6	-3.03	0.012
Magnitude	0.05	159.4	0.63	0.531	-0.11	58.7	-1.63	0.109
Direction AL	-0.17	291.2	-0.25	0.801	-0.21	291.3	-0.46	0.645
Direction L	-0.49	291.2	-0.74	0.459	-0.63	291.2	-1.38	0.168
Direction PL	-0.03	291.2	-0.05	0.962	-0.34	291.3	-0.74	0.458
Direction P	0.15	291.1	0.22	0.824	-0.48	291.2	-1.05	0.296
Direction PM	0.67	291.2	1.01	0.315	-0.47	291.3	-1.03	0.302
Direction M	-0.38	291.1	-0.58	0.565	-0.77	291.2	-1.69	0.092
Direction AM	-0.70	291.2	-1.06	0.289	-0.42	291.3	-0.91	0.364
Magnitude:Direction AL	0.17	291.9	1.39	0.166	-0.22	291.6	-2.66	0.008
Magnitude:Direction L	0.29	291.8	2.47	0.014	-0.15	291.6	-1.78	0.077
Magnitude:Direction PL	0.01	291.9	0.08	0.937	-0.06	291.6	-0.74	0.460
Magnitude:Direction P	-0.27	291.1	-2.27	0.024	0.14	291.2	1.70	0.090
Magnitude:Direction PM	-0.62	291.9	-5.22	<0.001	0.27	291.6	3.26	0.001
Magnitude:Direction M	-0.10	291.1	-0.87	0.387	0.36	291.2	4.43	<0.001
Magnitude:Direction AM	0.15	291.9	1.25	0.214	0.25	291.6	3.05	0.003
**Random effects**								
Std intercept	7.628				5.266			
Std PertMag	0.080				0.121			
Correlation	-0.10				-0.21			
**Model fit**								
R^2-marginal	0.03				0.04			
R^2-conditional	0.95				0.95			

Ankle inversion

	Ankle inversion moment trailing leg (Nm)				Ankle inversion moment leading leg (Nm)			
**Fixed effects**	**Parameter**	**df**	**t**	**p-value**	**Parameter**	**df**	**t**	**p-value**
Intercept	12.00	10.3	6.26	$<$0.001	-0.04	10.2	-0.03	0.973
Magnitude	0.06	160.7	1.26	0.211	0.00	76.3	-0.12	0.905
Direction AL	0.12	291.4	0.35	0.724	-0.01	291.2	-0.04	0.967
Direction L	0.17	291.3	0.49	0.626	0.07	291.1	0.35	0.724
Direction PL	0.31	291.4	0.88	0.378	0.10	291.2	0.51	0.613
Direction P	-0.01	291.2	-0.03	0.973	0.00	291.0	0.01	0.995
Direction PM	-0.02	291.4	-0.05	0.959	0.00	291.2	0.02	0.984
Direction M	-0.14	291.2	-0.39	0.696	-0.04	291.0	-0.18	0.856
Direction AM	-0.15	291.4	-0.43	0.667	-0.07	291.2	-0.34	0.735
Magnitude:Direction AL	-0.23	292.1	-3.71	$<$0.001	0.00	291.6	0.13	0.893
Magnitude:Direction L	-0.52	292.0	-8.42	$<$0.001	-0.06	291.6	-1.58	0.116
Magnitude:Direction PL	-0.52	292.1	-8.34	$<$0.001	-0.05	291.6	-1.25	0.211
Magnitude:Direction P	-0.13	291.2	-2.18	0.030	0.00	291.0	0.03	0.976
Magnitude:Direction PM	0.14	292.1	2.20	0.029	0.01	291.6	0.42	0.678
Magnitude:Direction M	0.28	291.2	4.57	$<$0.001	0.06	291.0	1.78	0.075
Magnitude:Direction AM	0.24	292.1	3.85	$<$0.001	0.06	291.6	1.65	0.101
**Random effects**								
Std intercept	6.017				3.759			
Std PertMag	0.042				0.045			
Correlation	0.63				-0.40			
**Model fit**								
R^2-marginal	0.06				0.00			
R^2-conditional	0.98				0.98			

Hip flexion

	Hip flexion moment trailing leg (Nm)				Hip flexion moment leading leg (Nm)			
**Fixed effects**	**Parameter**	**df**	**t**	**p-value**	**Parameter**	**df**	**t**	**p-value**
Intercept	16.50	10.3	6.30	<0.001	-19.00	11.0	-8.95	<0.001
Magnitude	0.02	44.5	0.29	0.776	-0.11	127.9	-1.21	0.230
Direction AL	-0.17	291.2	-0.37	0.714	0.15	291.2	0.21	0.837
Direction L	-0.75	291.2	-1.64	0.103	0.79	291.2	1.13	0.259
Direction PL	-0.51	291.2	-1.11	0.270	0.84	291.2	1.20	0.232
Direction P	-0.25	291.2	-0.55	0.581	0.08	291.1	0.12	0.908
Direction PM	-0.16	291.2	-0.36	0.721	-0.24	291.2	-0.34	0.737
Direction M	-0.07	291.2	-0.15	0.878	0.38	291.1	0.54	0.591
Direction AM	-0.08	291.2	-0.18	0.860	0.54	291.2	0.77	0.440
Magnitude:Direction AL	0.07	291.5	0.84	0.404	-0.04	291.8	-0.28	0.779
Magnitude:Direction L	0.54	291.5	6.60	<0.001	-0.54	291.8	-4.29	<0.001
Magnitude:Direction PL	0.59	291.5	7.11	<0.001	-0.50	291.8	-3.94	<0.001
Magnitude:Direction P	0.17	291.2	2.04	0.043	-0.03	291.1	-0.22	0.823
Magnitude:Direction PM	-0.06	291.5	-0.69	0.489	0.22	291.8	1.72	0.086
Magnitude:Direction M	-0.20	291.2	-2.47	0.014	-0.39	291.1	-3.14	0.002
Magnitude:Direction AM	-0.24	291.5	-2.88	0.004	-0.47	291.8	-3.76	<0.001
**Random effects**								
Std intercept	8.223				6.531			
Std PertMag	0.144				0.107			
Correlation	-0.15				-0.05			
**Model fit**								
R^2-marginal	0.03				0.06			
R^2-conditional	0.98				0.93			

Knee flexion

	Knee flexion moment trailing leg (Nm)				Knee flexion moment leading leg (Nm)			
**Fixed effects**	**Parameter**	**df**	**t**	**p-value**	**Parameter**	**df**	**t**	**p-value**
Intercept	10.80	10.3	3.12	0.010	0.42	11.0	0.20	0.844
Magnitude	-0.25	124.9	-2.87	0.005	-0.21	81.9	-2.20	0.031
Direction AL	-0.37	291.4	-0.56	0.576	-0.39	291.1	-0.57	0.567
Direction L	-1.33	291.4	-2.03	0.044	-0.15	291.1	-0.22	0.824
Direction PL	-1.03	291.4	-1.57	0.118	0.02	291.1	0.02	0.982
Direction P	-0.61	291.3	-0.93	0.351	-0.84	291.0	-1.24	0.215
Direction PM	-0.41	291.4	-0.62	0.535	-0.08	291.1	-0.11	0.909
Direction M	0.30	291.3	0.46	0.647	0.94	291.0	1.39	0.166
Direction AM	-0.10	291.4	-0.14	0.886	0.72	291.1	1.05	0.293
Magnitude:Direction AL	0.49	292.1	4.16	<0.001	0.68	291.6	5.59	<0.001
Magnitude:Direction L	1.47	292.0	12.42	<0.001	0.91	291.5	7.43	<0.001
Magnitude:Direction PL	1.57	292.1	13.23	<0.001	0.72	291.6	5.90	<0.001
Magnitude:Direction P	0.70	291.3	5.96	<0.001	0.18	291.0	1.51	0.131
Magnitude:Direction PM	0.10	292.1	0.83	0.409	-0.14	291.6	-1.15	0.253
Magnitude:Direction M	-0.37	291.3	-3.16	0.002	-1.10	291.0	-9.10	<0.001
Magnitude:Direction AM	-0.54	292.1	-4.57	<0.001	-0.99	291.6	-8.12	<0.001
**Random effects**								
Std intercept	10.838				6.463			
Std PertMag	0.104				0.146			
Correlation	-0.67				-0.24			
**Model fit**								
R^2-marginal	0.11				0.24			
R^2-conditional	0.98				0.94			

Ankle dorsiflexion

	Ankle dorsiflexion moment trailing leg (Nm)				Ankle dorsiflexion moment leading leg (Nm)			
**Fixed effects**	**Parameter**	**df**	**t**	**p-value**	**Parameter**	**df**	**t**	**p-value**
Intercept	-31.27	11.5	-14.93	<0.001	-8.07	10.9	-3.85	0.003
Magnitude	-0.35	177.0	-3.47	0.001	-0.35	59.7	-3.54	0.001
Direction AL	-1.01	291.6	-1.29	0.197	-0.01	291.0	-0.02	0.984
Direction L	-1.68	291.6	-2.16	0.031	-0.52	291.0	-0.79	0.431
Direction PL	-1.44	291.6	-1.85	0.065	-0.68	291.0	-1.02	0.309
Direction P	-0.66	291.5	-0.85	0.397	-0.17	291.0	-0.26	0.797
Direction PM	-0.18	291.6	-0.24	0.813	-0.25	291.0	-0.37	0.712
Direction M	0.76	291.5	0.98	0.330	0.98	291.0	1.48	0.139
Direction AM	1.24	291.6	1.59	0.112	1.04	291.0	1.56	0.119
Magnitude:Direction AL	0.96	292.4	6.84	<0.001	0.68	291.4	5.68	<0.001
Magnitude:Direction L	2.38	292.4	17.01	<0.001	1.43	291.4	12.08	<0.001
Magnitude:Direction PL	2.38	292.4	17.00	<0.001	1.34	291.4	11.31	<0.001
Magnitude:Direction P	0.87	291.5	6.30	<0.001	0.57	291.0	4.87	<0.001
Magnitude:Direction PM	-0.30	292.4	-2.14	0.033	0.27	291.4	2.30	0.022
Magnitude:Direction M	-1.38	291.5	-10.02	<0.001	-0.69	291.0	-5.88	<0.001
Magnitude:Direction AM	-1.29	292.4	-9.20	<0.001	-0.77	291.4	-6.49	<0.001
**Random effects**								
Std intercept	6.391				6.466			
Std PertMag	0.082				0.172			
Correlation	-0.79				0.05			
**Model fit**								
R^2-marginal	0.53				0.24			
R^2-conditional	0.95				0.95			
